# Overview of research on additive manufacturing of hydrogel-assisted lab-on-chip platforms for cell engineering applications in photodynamic therapy research

**DOI:** 10.1007/s00604-024-06683-9

**Published:** 2024-09-18

**Authors:** Adrianna Cieślak, Agnieszka Krakos, Julita Kulbacka, Jerzy Detyna

**Affiliations:** 1 Department of Mechanics, Materials and Biomedical Engineering, Faculty of Mechanical Engineering, Wrocław University of Science and Technology, Wrocław, Poland; 2 Department of Microsystems, Faculty of Electronics, Photonics and Microsystems, Wrocław University of Science and Technology, Wrocław, Poland; 3 Department of Molecular and Cellular Biology, Faculty of Pharmacy, Wroclaw Medical University, Wroclaw, Poland; 4 Department of Immunology and Bioelectrochemistry, State Research Institute Centre for Innovative Medicine, Vilnius, Lithuania

**Keywords:** Lab-on-chip, Hydrogel, Additive manufacturing, 3D printing, Photodynamic therapy, Cancer cells

## Abstract

Lab-on-chips supported by hydrogel matrices are excellent solutions for cell culture; thus, this literature review presents examples of scientific research in this area. Several works are presenting the properties of biocompatible hydrogels that mimic the cellular environment published recently. Hydrogels can also be treated as cell transporters or as a structural component of microfluidic devices. The rapidly growing scientific sector of hydrogel additive manufacturing is also described herein, with attention paid to the appropriate mechanical and biological properties of the inks used to extrude the material, specifically for biomedical purposes. The paper focuses on protocols employed for additive manufacturing, e.g., 3D printing parameters, calibration, ink preparation, crosslinking processes, etc. The authors also mention potential problems concerning manufacturing processes and offer example solutions. As the novel trend for hydrogels enriched with several biocompatible additives has recently risen, the article presents examples of the use of high-quality carbon nanotubes in hydrogel research enhancing biocompatibility, mechanical stability, and cell viability. Moving forward, the article points out the high applicability of the hydrogel-assisted microfluidic platforms used for cancer research, especially for photodynamic therapy (PDT). This innovative treatment strategy can be investigated directly on the chip, which was first proposed by Jędrych E. et al. in 2011. Summarizing, this literature review highlights recent developments in the additive manufacturing of microfluidic devices supported by hydrogels, toward reliable cell culture experiments with a view to PDT research. This paper gathers the current knowledge in these intriguing and fast-growing research paths.

**Graphical abstract:**

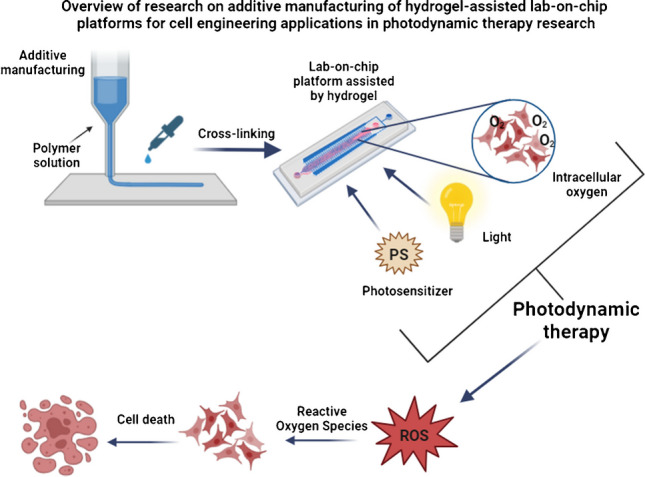

## Introduction

There is an apparent increase in the incidence of cancer worldwide. According to 2020, more than 19 million cases of cancer have been diagnosed globally [1]. Due to the rapidly increasing incidence of cancer, the scientific world is focused on developing increasingly innovative cancer research methods. Combining knowledge and skills in biomedicine and engineering is the key to achieving this development. Innovative methods make it possible to raise the standards of health care through more accurate and controlled research. This dissertation focuses on microfluidic devices provided in the form of lab-on-chip (LOC) solutions used for cell engineering investigation. Fabrication methods, especially additive manufacturing, as well as materials, with a view to hydrogels, will be described thoroughly to review the applicability of LOCs in cell culturing research. Current state-of-the-art concerning photodynamic therapy (PDT), as a promising and noninvasive anticancer therapy to be evaluated directly on hydrogel-assisted LOCs, will be presented in detail. The following topics will be described in the relevant sections of the paper: hydrogel additive manufacturing techniques, 3D printing of hydrogel for cell engineering, lab-on-chip assisted by hydrogels in cell engineering, carbon nanotube (CNT)-doped hydrogels, and photodynamic therapies on-chip. These various topics merge to form a new approach to the development and evaluation of anticancer therapies.

Lab-on-chips are innovative microlaboratories like integrated analytical microsystems, used, among other things, in cell or tissue engineering in vitro. The main advantage of such biosystems is the ability to simultaneously culture and analyze cells in real-time, bypassing the cell-killing step. In addition, by minimizing such systems to the microscale, production and culture costs can be reduced, and thus, biological and chemical wastes are also reduced, minimizing environmental pollution. Lab-on-chips are also portable and easy to handle and transport. Lab-on-chip fabrication materials are usually glasses and polymers (the most common is poly(dimethylsiloxane) PDMS [2, 3]). Apart from the standard microengineering techniques like photolithography, wet etching and bonding, additive manufacturing techniques such as extrusion-based printing, jetting-based printing, and vat photopolymerization-based printing are becoming more and more popular. The structure of LOCs is distinguished by characteristic tubules, chambers, and channels in the microsize, allowing microfluidics to control the cellular environment [4]. These types of microchannels are intended to imitate biological capillaries [5].

Hydrogels are spatially cross-linked materials composed of hydrophilic synthetic or natural polymers. They can absorb sizable amounts of liquid while maintaining a three-dimensional structure, thus, hydrogel materials can swell in water or body fluids. The liquid in hydrogels is the medium that allows the diffusion of substances, while the degree of cross-linking of the hydrogel polymer is responsible for the efficiency of their transport. The polymer chains that form the hydrogel network can be chemical (irreversible) or physical (reversible) in nature. The mechanical properties of hydrogels depend primarily on the concentration of ingredients or the degree of crosslinking, i.e., the time, concentration, and type of crosslinking agent. Crosslinking of a polymer solution is a process that leads to a hydrogel gaining stability in geometry and structure, and increasing its mechanical strength. Studies conducted on hydrogels include analysis of hydrogel pore size, cross-linking and mechanical strength, viscosity, degree of swelling, and other rheological properties [6–13]. It is common practice to enrich hydrogels with various additives. An example known in the literature, but not so common, is the use of biocompatible carbon nanotubes (CNTs), which are one of the allotropic varieties of carbon. Single-walled CNTs (SWCNTs) and multi-walled CNTs (MWCNT) are distinguished. Both kinds of additives are used in the biomedical field. CNTs are so special because they can imitate natural collagen material because of their similar form and dimensions [14].

**Fig. 1:**
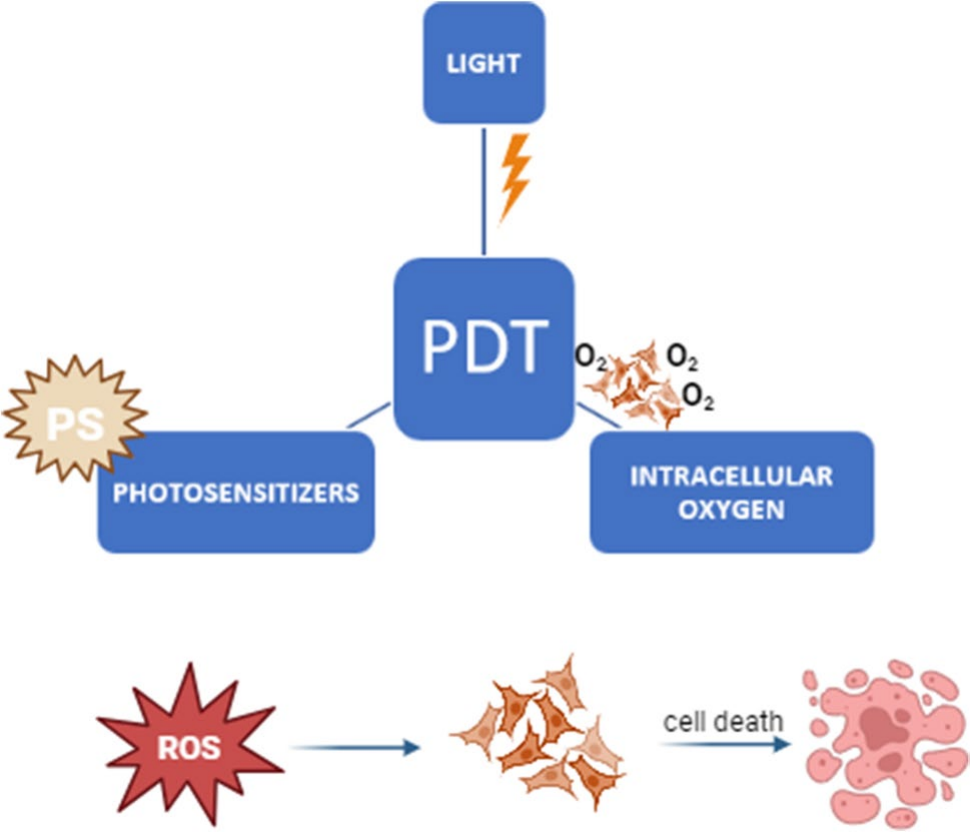
Photodynamic therapy (PDT)—mechanism of action [73]

As mentioned earlier in this paper, one of the current challenges in the scientific world is the development of new cancer therapies. For this reason, many researchers have recently focused on a relatively new, non-invasive treatment strategy—photodynamic therapy. Photodynamic therapy is a local treatment of oncological cancers (head, lung, skin) and non-oncological diseases (lichen sclerosis). Photodynamic therapies can be the main treatment pathway or can be combined with other known solutions, such as radiotherapy or chemotherapy. The PDT mechanism involves using light-sensitive compounds known as photosensitizers (PS), which are selectively accumulated in tumor cells and destructive only for them. The effectiveness of photodynamic therapies is determined by the presence of intracellular oxygen and light absorbed by such PS (Fig. 1). Phototoxic reactions are initiated by activating the mentioned photosensitizer (accumulated in cancer cells) with light of a given wavelength and power. Next, the generation of reactive oxygen forms (ROFs) occurs, which have toxic effects on cell growth by, among other things, the occurrence of so-called oxidative stress [15, 16].

Based on all of this, the purpose of this literature review is to highlight the recent achievements in the fields of additive manufacturing of microfluidic devices supported by hydrogels (bare and CNT-doped) for cell engineering, towards research on PDT, as a modern anti-cancer therapy, investigated directly on-chip. According to the best authors’ knowledge, this is the first review paper in the interesting fields, focusing on the mentioned subjects as a whole. It is possible to read about the issues separately, for example [13, 17–20].

## Hydrogel additive manufacturing techniques

The use of additive manufacturing to fabricate hydrogels, as well as entire LOC constructs, is becoming increasingly common. Additive manufacturing involves the direct fabrication of objects in a single process by depositing various materials. As a result, raw material compositions can be used more efficiently, resulting in less waste and less material utilization. This feature provides several benefits, particularly for small-scale production or custom approaches, including shorter production times and lower costs. In biomedicine, the use of AM technology is of great importance. One example is the microscale manufacturing of microfluidic devices and 3D printing with hydrogels for cell or tissue engineering applications. This advanced technology facilitates the development of novel solutions that are often too complicated or cannot be manufactured using traditional methods. The ability to use AM allows for the continuous development of these technologies, but also the evolution of biomedicine. There are several AM techniques used in biomedicine, and their choice largely depends on the specific application, the material used, and the requirements imposed on the final component. The components and by-products produced must be biocompatible (no destructive effects on living cells). Also important is the durability of the 3D print under specific conditions, such as the cell environment, external and internal forces, stress formation, or corrosion. Manufactured parts must be produced with high precision and accuracy to fit the computer-designed 3D model [21]. Regarding this literature review, technologies for AM-fabricated microscale lab-on-chip devices with hydrogel structures will be presented exclusively.

According to ASTM standards, three main techniques for additive manufacturing of hydrogels can be mentioned, such as extrusion-based 3D printing, jetting-based 3D printing, and vat photopolymerization-based 3D printing.

Ink extrusion is carried out mainly by pneumatic pressure, mechanical compression, or a solenoid. The 3D-printed object is formed layer-by-layer, and the extruded fiber is characterized by a diameter in the range of 150–300 μm. The literature overview shows that this is the most common 3D printing process. This technology is characterized by low process costs, a wide range of available materials, and the availability of dedicated 3D printers. The weaknesses of extrusion technology include low resolution and printing speed, as well as clogging of the printing nozzle. In addition, a cell-killing phenomenon can occur due to the shear stresses created during the extrusion of the bioink (ink with cells). In summary, it provides a lower cell survival rate compared to other 3D bioprinting methods [13, 17, 22].

Jetting-based 3D printing, in turn, is a less popular AM technology in which ink is formed into droplets. Ink droplets are carried out mostly by acoustic and thermal methods. This technology has better resolution and cell survival compared to extrusion processes. However, there are fewer commercial inks and inkjet 3D printers suitable for this technology available. Droplet ink leads to less efficient additive manufacturing. Moreover, the hydrogel inks should have a relatively low viscosity to avoid clogging the printing head. Thus, the effects of 3D bioprinting utilizing this method and the subsequent biological activity of cells are still not well-known [23, 24]. Lastly, jetting-based 3D printing was performed in the research of Negro A. et al. in 2018 [25] and Walczak R. et al. in 2018 [26].

It is also necessary to consider ink requirements and the impact of bioprinting parameters, as well as bioink properties on cell viability from the jetting-based bioprinting point of view. Ng et al. (Ng 2021) conducted research using a drop-on-demand material jetting technique and a thermal 3D printer. The results were presented by evaluation of surface tension, density, and viscosity experiments, among others. The authors proved that the increase in cell concentration (primary human dermal fibroblasts, 4 mln cells/ml) resulted in a slower droplet impact speed (optimal 5.77 m/s) when spraying droplets containing sub-nanoliter volume cells. Reducing the droplet impact velocity led to higher cell viability and improved 3D print quality. Moreover, the authors highlighted that printing speed was crucial regarding cellular viability. Each of the layers was 3D bioprinted within two minutes to prevent excessive droplet evaporation, which negatively affects cell survival rate (hypertonic environment occurring, causing fibroblast apoptosis). Moreover, the higher the cell concentration, the higher the values of density and viscosity of the bioinks, and the lower the surface tension. The study confirmed that controlling droplet impact velocity and droplet volume led to high short-term (1 day) fibroblast viability and long-term (7 days) proliferation of 3D-printed cells [27]. A slightly older study of the same scientific team (Ng 2017) investigated the influence of synthetic bioink properties on 3D bioprinting and cell activity during that process. They prepared five different concentrations of polyvinylpyrrolidone (PVP)-based bioink and also characterized their density, viscosity, and surface tension. The authors found that the viscosity and density of PVP-based bioinks increased with rising substance concentration. In contrast, the surface tension of the bioink decreased as its concentration increased. Printing parameters included, among other things, a constant pressure of 25 kPa, since above this value—shear stress has been shown to hurt living cells contained in the bioink. Other findings were that the viscosity of the bioink increased with increasing cell concentration (0.5–2.5 mln cells/ml), and also reduced the surface tension due to a decrease in the total free energy of the bioink. Cell viability is strongly influenced by the Z-factor, defined as the inverse of the Ohnesorge number, meaning the ratio of the Reynolds number to the square root of the Weber number; independent of the bioink velocity. During the research, the following Z-factor range of values was obtained: 5.75 ≤ Z ≤ 64.36. According to the performed experiments, printable bioink with a low Z-value (< 9.3) is suitable for achieving high cell viability (> 90%) [28].

Laser-assisted 3D (bio)printing (also known as Laser-Induced Forward Transfer—LIFT) belongs to the group of jetting-based printing technology. It is another layer-by-layer process using a laser beam of appropriate power (tiny droplets are ejected during the 3D printing process using the laser source). The process singles out the ink and the photoabsorbent material just above it. Laser-assisted 3D printing is the most expensive and complicated method among those mentioned above. The 3D-printed parts have low mechanical stability, but due to the lack of a printing nozzle, there is no problem with printing head clogging or shear stress (a major benefit of this technology concerning bioinks). Moreover, laser 3D printing shows the highest resolution, precision, and cell viability. However, ink viscosity, layer thickness, and laser parameters are crucial in laser AM [23, 24]. There are several biomaterials and biological objects that can be processed using this technique. The sample research using this technology was carried out by Kingsley D. et al. in 2019 [29]. Various parameters affect cell viability during LIFT processes, for example, laser parameters such as wavelength. The laser wavelength should be carefully selected. Typically, near-infrared or UV lasers are used to minimize thermal damage to cells by reducing the interaction with biological tissues. Another parameter is pulse duration—shorter pulse durations (femto/pico-seconds) may reduce the heat-affected zone, thus minimizing thermal damage to cells. However, excessively short pulses can lead to high peak intensities that may cause photomechanical damage. It should be highlighted that the viscosity of the bioink affects the formation of the jet or droplet during the LIFT process (bioprinting). Higher viscosity may require higher laser energy to induce transfer, potentially increasing the risk of cell thermal damage. Crucial is choosing the cell type during experiments. Different cell types have varying levels of tolerance to the mechanical and thermal stresses induced by LIFT. For example, some stem cells or primary cells may be more sensitive compared to immortalized cell lines. Selecting a laser parameter set that matches the tolerance level of the specific cell type being printed is essential [30–33].

The last technique, vat photopolymerization-based 3D printing, is a method that does not use a nozzle. In this case, the inks must exhibit sensitivity to light, which leads to their polymerization, thus giving structural stability to the 3D prints. The light source can be UV radiation. The most common form of vat photopolymerization is stereolithography, which presents low cost, a short printing time, and a relatively high resolution. Examples of photo-initiators used in stereolithography are complex substances of lithium phenyl-2,4,6-trimethylbenzoylphosphinate (LAP, 0.5% w/v) and commercially available Irgacure 2959 (1-[4-(2-hydroxyethoxy)-phenyl]-2-hydroxy-2-methyl-1-propanone) [34, 35], Irgacure 819 (phenylbis(2,4,6-trimethylbenzoyl)phosphine oxide) and diphenyl(2,4,6-trimethylbenzoyl)phosphine oxide (TPO, 3% w/v relative to the polymer) [36]. The materials used in this technology are light-curing resins (commercial e.g. Dental SG and Dental LT Clear or self-made). The safe parameters of UV light (LED) from the cell viability point of view are as follows – 5 mW cm^−2^ at 365 nm and 30 mW cm^−2^ at 405 nm, UV exposure time: 15 min. [37], or 20 mW cm^−2^ with radiation 30 s [36]. Interestingly, this method allows the processing of inks with higher viscosity. There is also no problem with clogging or shear stress. Unfortunately, the main limitation of stereolithography is the poor choice of light-sensitive and biocompatible materials at the same time [23, 24]. Interesting stereolithography applications were presented in the research of Xue D. et al. in 2018 [34] and Piironen K. et al. in 2020 [37].

## 3D printing of hydrogel for cell engineering

**Fig. 2:**
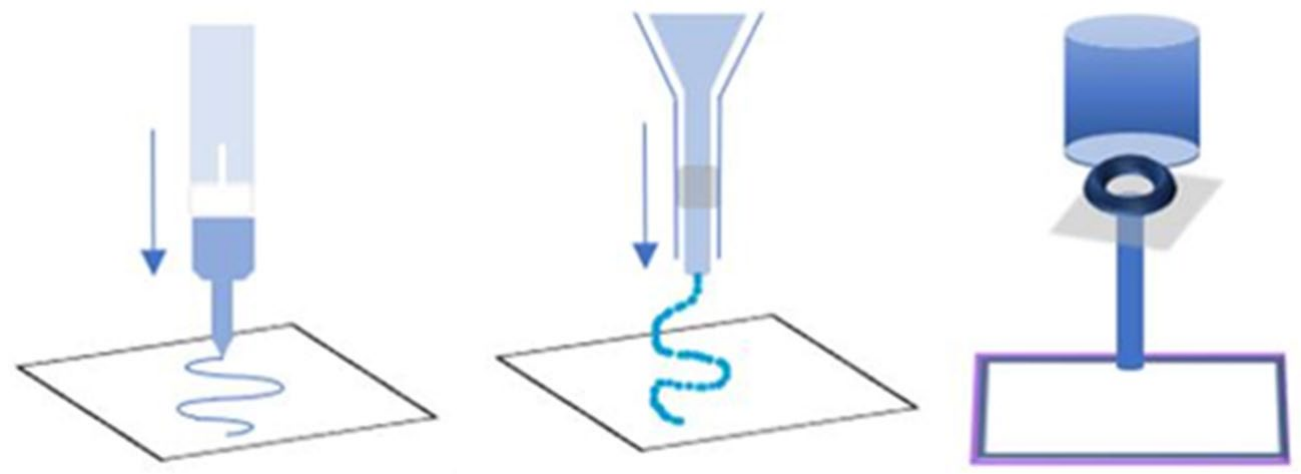
Visualization of the main AM technologies dedicated to hydrogels—from the left: extrusion-based 3D printing, jetting-based 3D printing, and vat photopolymerization-based 3D printing (Own source)

A variety of natural and synthetic inks are used to obtain stable hydrogel structures through 3D bioprinting or 3D printing and post-processing (e.g., cross-linking method). The inks should exhibit adequate rheological properties to enable the printability of the material, and the hydrogels themselves must exhibit satisfactory mechanical and biological properties concerning the strength of the hydrogel matrix to ensure cell culturing [38]. As mentioned earlier, in 3D printing, the following AM technologies are distinguished: extrusion-based 3D printing, jetting-based 3D printing, and vat photopolymerization-based 3D printing, Fig. 2.

The specification of the AM technologies is presented in Table 1 [22]. The base of the AM process is to select an appropriate ink, that is, a printable biomaterial, which may contain living cells in its composition (then bioink). Hydrogels are excellent bioinks because of their extracellular matrix-like properties [20, 39].

**Table 1:** The main differences between AM technologies used in 3D printing and bioprinting of hydrogels (compiled based on [22])

Specifications	Extrusion	Jetting-based 3D printing	Stereolithography
Resolution [µm]	**100**	10—50	0.2—6
Cell viability [%]	**4—95**	85	> 85
Cell density [-]	**High**	Low	Medium
Material viscosity [mPa/s]	**6 × 10**^**7**^	3.5—12	Unlimited
3D printing speed [-]	**Slow**	Quick	Quick
Process costs [-]	**Medium**	Low	Low

**Fig. 3:**
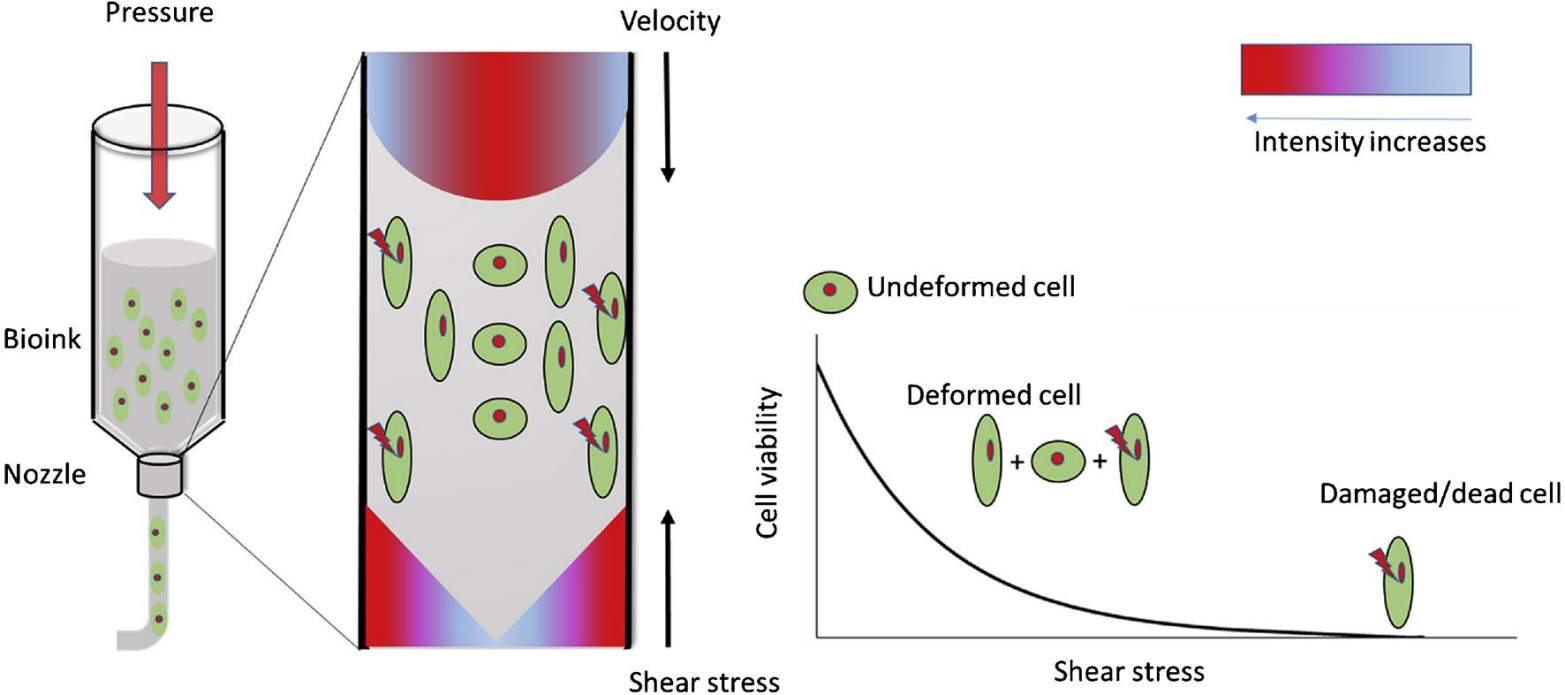
The effect of shear stress on cells during extrusion-based 3D bioprinting [13]

The impact of extrusion-based bioprinting processes on cell activity requires greater attention. More precisely, 3D bioprinting can affect not only cell death but also cell growth, differentiation, or shape. The bioink applied in 3D bioprinting is a unique ink composition containing living cells. Thus, the AM process is characterized by the extrusion of biological cells immersed in biocompatible ink (natural or synthetic). In that dynamic procedure, cells are exposed to a variety of interactions, especially mechanical ones, which might be fatal. That is why 3D bioprinting is such a specific and demanding process. Whether cells survive the 3D bioprinting process corresponds to the functionality of the designed structure, including tissues, biosensors, and scaffolds, among others. Certainly, the 3D bioprinting process is a more expensive procedure than standard ink extrusion, and this is important to emphasize. In addition, the preparation and establishment of parameters in 3D bioprinting is more complex and time-consuming. One of the most serious problems faced during bioprinting is the formation of inappropriate shear stress values, which destructively affect the living cells contained in the bioink. Attention to this issue is drawn by Boularaoui S. et al. (Boularaoui 2020). In the process of extrusion of bioink by pneumatic method, pressure is applied to the extruded material. The narrower the diameter of the nozzle, the greater the pressure that must be applied to the bioink. The most vulnerable cells are those near the walls of the printing nozzle. Therefore, cells within the core of the extruded fiber are more secure, as shown in Fig. 3. Furthermore, the shear stress created in the 3D bioprinting process is more deadly the higher the values it reaches. The lethal effect of the induced shear stress is determined by the printing parameters and the properties of the bioink (biomaterial composition, cell type and concentration, etc.). Shear stresses generated during bioprinting may reach values of up to 60 kPa at high 3D printing speeds. In the context of 3D bioprinting by extrusion, the authors paid attention to the bioink (its composition, properties, concentration, etc.) as well as the 3D printing parameters and related consequences. The phenomena occurring in the printing nozzle filled with bioink were highlighted. Namely, how the choice of materials and 3D printer parameters could affect the printing process and the biological activity of the cells contained in the bioink [13]. The described research needs optimizing bioink formulations and printing parameters, developing better bioreactor systems for post-printing tissue maturation, and exploring new materials that mimic the ECM.

**Fig. 4:**
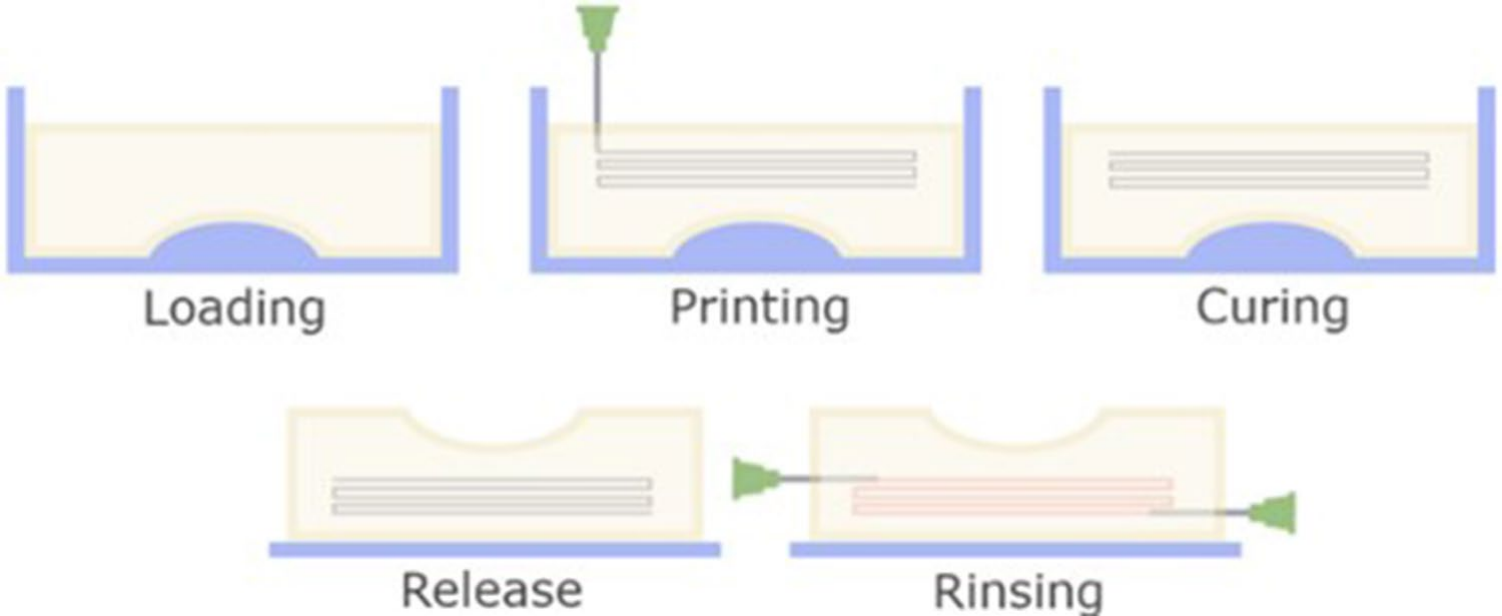
Scheme of fabrication of a hydrogel microfluidic device [40]

Stumberger G. and Vihar B. (Stumberger 2018) described the additive manufacturing of hydrogels and developed perfused microfluidics within hydrogel matrices. In the study, the FRESH method was used to deposit the material inside a matrix (gelatin hydrogel) that supports the printed ink, allowing any geometry to be made. The hydrogel matrix was enriched with cross-linking calcium ions so the microchannel-forming material could polymerize when applied to the matrix. Once the channel-making process was completed, the matrix could be removed by heating and liquefying the material. The fabrication procedure involved placing the gelatinous suspension in a mold and then 3D printing the channel path. The matrix was then solidified so that the structure became stable and could be removed from the mold, and the support material could also be removed. The stages of manufacturing the hydrogel matrix are shown in Fig. 4. Based on this study, the following observations were made: smaller gelatin grains in the matrix provided better microchannel resolution and shape fidelity. In contrast, larger grains caused a structure to be more stable. According to the Authors, the optimal grain size was in the range of 90–200 µm. To test the versatility of the fabrication technique, an alginate matrix was prepared and tested, showing positive test results. Thus, potential applications of this research cover, e.g., the development of invisible vascular tissues – vascular grafts [40]. Although the article demonstrated the feasibility of creating microfluidic structures within hydrogels, it did not thoroughly address the scalability of the technique for large-scale production. Moreover, the long-term stability and durability of the embedded microfluidic channels within the hydrogel matrices were not extensively discussed. Future research in this area should focus primarily on scalability and long-term stability to develop this technology into real-world applications.

**Fig. 5:**
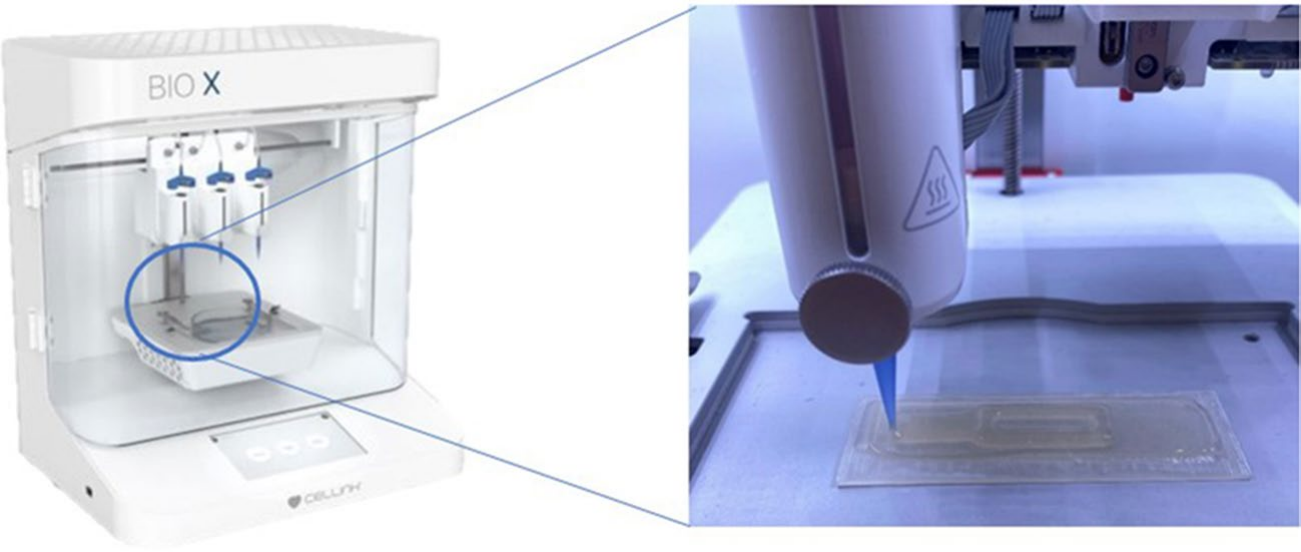
The BIO X 3D printer from CELLINK. Extrusion 3D printing process (Own source)

Another research by Chrenek J. et al. (Chrenek 2022) presented the tissue bioprinting protocol employed for the BIO X 3D printer from the CELLINK company, as shown in Fig. 5. The authors checked application possibilities for that 3D printer and commercial hydrogel ink based on fibrine, self-made hydrogel consisting of alginate (2%) and gelatin (2%) (high viscosity), as well as hydrogel bioink containing living mesenchymal cells were used. The scientists proposed, i.e., an agarose support bath to increase the stability of the hydrogel structures printed utilizing commercial, low-viscosity ink. The protocols were provided thoroughly, e.g., calibration, printing parameters, ink preparation, and cross-linking methodology (solution, concentration, time, etc.) were described. Moreover, scientists indicated the maximum pressure of the 3D printing process to be 200 kPa. This parameter is crucial, especially for cell viability, as too much pressure could kill the cells. According to the article, the appropriate pressures are 11 kPa for commercial ink and 20 kPa for alginate-gelatin ink. Selected 3D printing parameters indicated in this paper are shown in Table 2.

**Table 2:** 3D printing parameters using different hydrogels [41]

Parameter	Petri dishes	With an agarose bath	Alginate-gelatine hydrogel
3D printing speed [mm/s]	15	15	20
Calibration height [mm]	0.1	1	0.1
Pressure value [kPa]	11	11	20

The authors also mentioned some potential problems that could occur during 3D printing and proposed interesting solutions. For instance, the formation of air bubbles could be minimized by slowly stirring with the tip of the pipet or centrifugation of the gel in a laboratory centrifuge. The missing or inconsistent extrusion of material can be offset by increasing the printing pressure, replacing the print head tip if it is clogged or damaged, or filling the print syringe with a new gel. If there is a problem with a clogged nozzle tip, it is worth removing the hydrogel clot by increasing the pressure in the printer or manually removing it and moving the print tip away from the work table to prevent possible cross-linking of the ink. To eliminate the problem of maintaining the structure shape after printing, the cross-linking agent should be applied to the substrate for a few minutes and then removed. Another possibility is to extend the cross-linking time of the ink. If there is a problem with a leaking nozzle tip between prints, the pressure in the 3D printer should be reduced, or just the printing tip should be wiped [41]. Despite the innovative approach in this article, several limitations must be considered. Namely, technical challenges – print resolution focused on the pneumatic printhead of the BIO X 3D printer. Achieving the fine spatial precision needed for intricate neural networks could be difficult, potentially impacting the fidelity of printed tissues compared to natural neural structures​. One of the major challenges in tissue engineering is providing sufficient vascularization for nutrient and oxygen delivery. Among other things, the presented protocol did not take into account what could limit the size and durability of the printed neural tissues.

Another interesting work of Tarassoli S. et al. (Tarassoli 2021) presented a systematic review concerning bioinks for 3D bioprinting. Eligibility for inclusion of articles in this paper was as follows: focusing on extrusion 3D bioprinting, testing animal or human cells, in vitro, as well as in vivo tests, research articles, and English language articles only. Natural inks, synthetic inks, and inks resulting from a combination of the two were distinguished. According to the paper, the most common hydrogels used as inks were alginates, polycaprolactone (PCL, synthetic), gelatin, and methacrylate gelatin. The others included, e.g., chitosan, collagen, gellan gum, or Matrigel. The average resolution of the bioprinting process for natural bioinks was defined as 1 mm and 0.5 mm for synthetic bioinks (Table 3). According to the authors, the lowest resolution obtained was 0.025 mm [20].

**Table 3:** Resolution values during 3D bioprinting processes for specific hydrogels [20]

Hydrogel	Resolution value [mm]
Alginate	0.3
PCL	0.1–1
Gelatin	0.15–0.8
Methacrylate gelatin	0.1–0.5
Chitosan	0.1–0.8
Collagen	0.15–0.8
Gellan gum	0.25–0.8
Matrigel	0.2

From the review article’s point of view, the reviews might predominantly include studies that report positive outcomes. Negative results are less likely to be published. This bias can skew the understanding of bioink effectiveness and applicability. Moreover, review articles may emphasize bioinks that are already well-documented, potentially neglecting innovative or experimental bioinks that could offer unique properties and benefits. Furthermore, review articles usually compare results (e.g., biological, mechanical, and others) obtained with different equipment and even laboratory methods, which can influence possible inconsistencies.

**Fig. 6:**
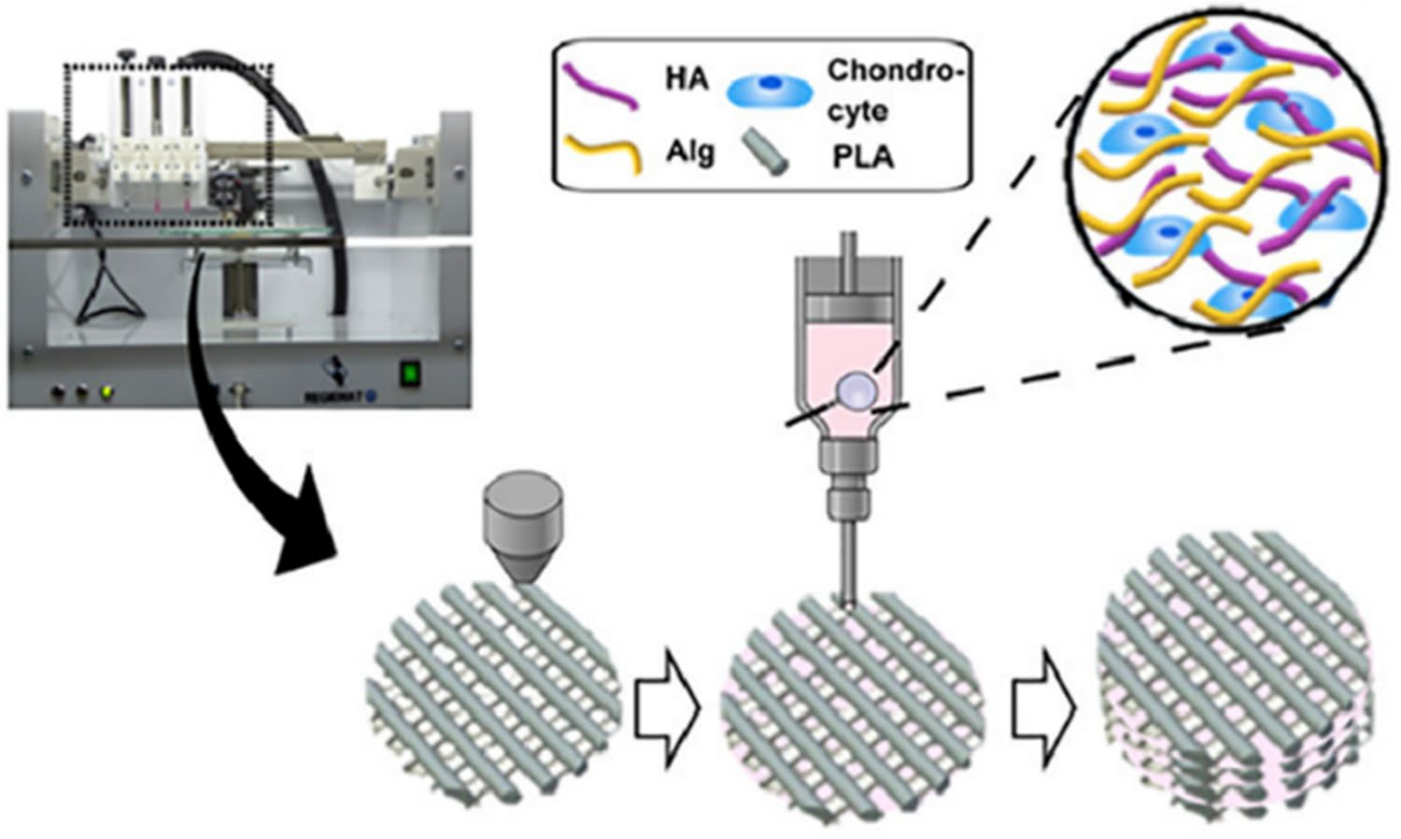
Scheme of 3D bioprinting process [42]

In the paper of Antich C. et al. (Antich 2020), the development of a hydrogel bioink based on hyaluronic acid (1%) and sodium alginate (2%) with the potential to manufacture articular cartilage additively was presented. The tests performed were in vitro type. The cell scaffold was based on a synthetic polylactide (PLA), into which pores the bioink was imprinted. Calcium ions (Ca^2+^) were used as the cross-linking agent to obtain the hydrogel. The additive manufacturing of PLA was performed using the extrusion of the bioink, as shown in Fig. 6. Next, a mixture of hydrogel and human articular chondrocyte cells was applied to a scaffold. To verify the solution durability and investigate the degradation of hydrogels in the cellular environment in vitro, obtained hydrogel constructs were mechanically validated at first. For this purpose, compression tests and shear tests were performed. The research results indicated that the designed hydrogels showed a higher modulus of elasticity compared to the control groups (PLA + sodium alginate and pure PLA). This may be related to the viscoelastic properties of the hydrogel based on hyaluronic acid and alginate (the ability to transfer loads). When it comes to biological research, it was focused on cell viability, proliferation, and selected biological activities of the cells. Live/dead assays were performed before and after the 3D bioprinting processes. Based on the tests, it was shown that 3D bioprinting of the designed scaffolds allowed for the regeneration of joint cartilage. Therefore, the scaffolds promoted chondrogenesis, i.e. the process of cartilage formation. According to the authors, a hydrogel consisting of alginate and hyaluronic acid dissolved in deionized water was a suitable biomaterial for use in 3D bioprinting for such biomedical applications [42]. Nevertheless, the research referred to a specific type of cells and hydrogels. The authors did not report on the possibility of extending the research to other materials or cells, which affects the versatility of the described research. The discrepancy in the properties of 3D-printed tissues and natural tissues can affect the performance and durability of bioprinted constructs under physiological conditions. However, the data obtained allows for further steps and provides opportunities to perform in vivo tests and their real use.

**Fig. 7:**
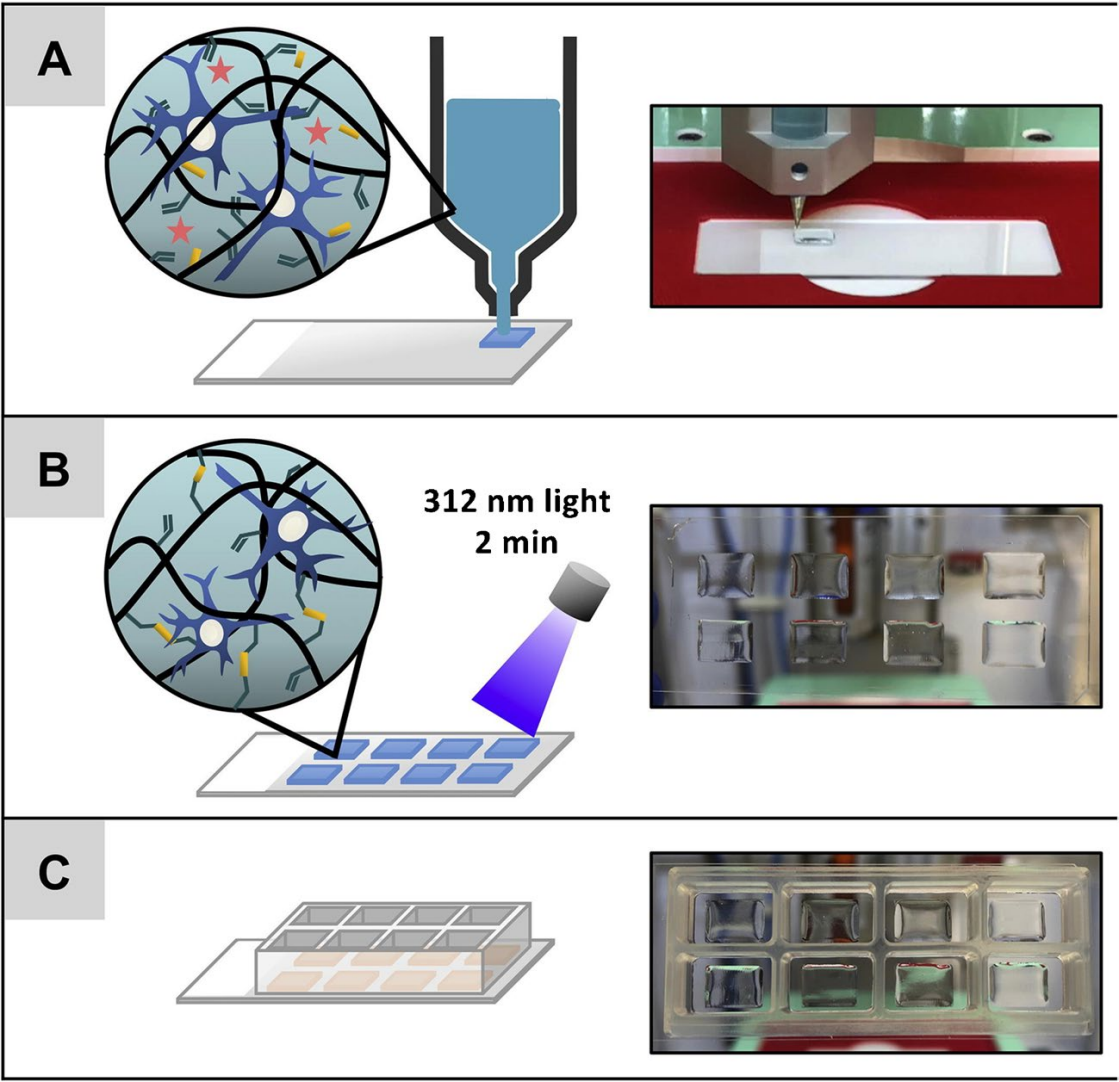
Scheme of biofabrication steps: **a**) 3D bioprinting, **b**) cross-linking, **c**) mounting of silicone cover [43]

In turn, Kiyotake E. et al. (Kiyotake 2019) developed a pentanoate-modified hyaluronic acid hydrogel, as a new biomaterial constituting a bioink in AM and, more specifically, in extrusion processes. The Authors prepared several different hydrogels that differed in concentration of hyaluronic acid. The bioink was a mixture of hydrogel stem cells and nerve cells (two different types separately). Cross-linking processes were performed using ultraviolet (UV) light. The biofabrication process is presented in Fig. 7. The scientists characterized the rheological properties of prepared hydrogels, such as viscosity, yield strength, and recovery modulus of storage. These parameters are inherent in the printability of the material. According to rheological research results, the behavior of viscosity informed about the bioink thinning properties under the influence of shear during 3D bioprinting. In turn, the parameter of the yield strength was crucial to maintain the fidelity of the shape to the designed model. The authors found that cell concentration had a negligible effect on the printability of the bioink. In addition, cell viability after the 3D bioprinting process was satisfactory for the two types of cells tested. Nevertheless, the authors pointed out the need for further research focused on, among others, cell concentration or sterilization methods of the bioink. Although the scientists referred to a specific hydrogel (hyaluronic acid enriched with pentanoate), the versatility of rheological research methods gives the possibility to extend the studies to other bioinks. The study of additional biomaterials would strengthen the validity and correctness of the research. Scientists made a qualitative assessment of shape fidelity, but to obtain reliable results, a quantitative analysis should also be performed, e.g., by the use of dedicated programs analyzing, among others, the size or number of hydrogel pores. In conclusion, the authors emphasized the need to focus on standardizing bioink and 3D bioprinting parameters, not only on a trial-and-error approach [43]. The study focused on three specific rheological parameters (yield stress, viscosity, and storage modulus recovery) to assess printability. While these are critical, other factors such as thermal stability or long-term mechanical stability were not thoroughly examined. According to biological experiments, the study reported minimal impact on printability with increasing cell concentrations but did not extensively evaluate the long-term viability and functionality of encapsulated cells after 3D printing. To ensure that the bioink supports cell proliferation and differentiation, detailed biological assessments are necessary.

The next research was described by Shi Y. et al. (Shi 2018), who presented a bioink based on methacrylate gelatin (5%) and collagen (8%) with the addition of tyrosinase. Different concentrations of tyrosinase: 0, 100, 300, 500, and 800 u/ml were applied to achieve the 3D bioprint skin-like structures with living cells (human melanocytes, keratinocytes, and dermal fibroblasts). The 3D bioprinting was performed using the bioink extrusion. The cross-linking process was performed in two steps by tyrosinase enzyme and UV light. An innovation in these studies was the use of tyrosinase, which increased the stability of the hydrogel and gave the effect of skin color. According to the results of the biological tests, all cells were characterized by a high percentage of viability, exceeding 90%. The presence of tyrosinase reduced the growth and mobility of fibroblasts, increased the proliferation of melanocyte cells, and was neutral for keratinocytes. Moreover, in vivo tests showed that the addition of tyrosinase shortened the healing time of wounds and, more specifically, the reconstruction of the damaged epidermis and dermis [44]. Although the study reported high initial cell viability (> 90%) for human melanocytes, keratinocytes, and dermal fibroblasts, the long-term viability and functionality of these cells within the bioprinted constructs were not thoroughly investigated. Moreover, from the mechanical point of view, long-term stability and degradation rates of bioink are also important factors that were not fully addressed.

Another study conducted by Jain T. et al. (Jain 2021) was based on the evaluation of the 3D bioprinting process when incorporating the L929 mouse fibroblast cell line into a gelatine methacrylate (GelMA, 10% w/v) ink at different concentrations: 1, 5, and 10 × 10^6^ cells /ml. The AM process was based on 3D bioprinting by extrusion of the bioink. The cross-linking process was performed using UV light. The motivation for the study was the instability of the physicochemical properties of bioink dedicated to 3D bioprinting. The scientists performed mechanical-rheological tests and evaluated viscosity variation, shape fidelity, and compressive strength of 3D prints. It was shown that when the concentration of cells in the bioink increased, the homogeneity of their distribution in the hydrogel was improved. Furthermore, GelMA ink (in the concentration range of 7–15% w/v) was found to be suitable for biomedical applications with cells directly incorporated into this biomaterial or separately. To sum up, the addition of cells in the bioink did not statistically significantly affect the rheological properties of the biomaterial or the 3D printing process in this study [45]. The question is what if higher concentrations of cells or other cell types were used? Normalizing the standards for the 3D printing of biomedical products for in vitro and in vivo applications is the major issue. Furthermore, the study did not extensively examine long-term cell viability and functioning within 3D-printed constructs, which is crucial for practical applications.

**Fig. 8:**
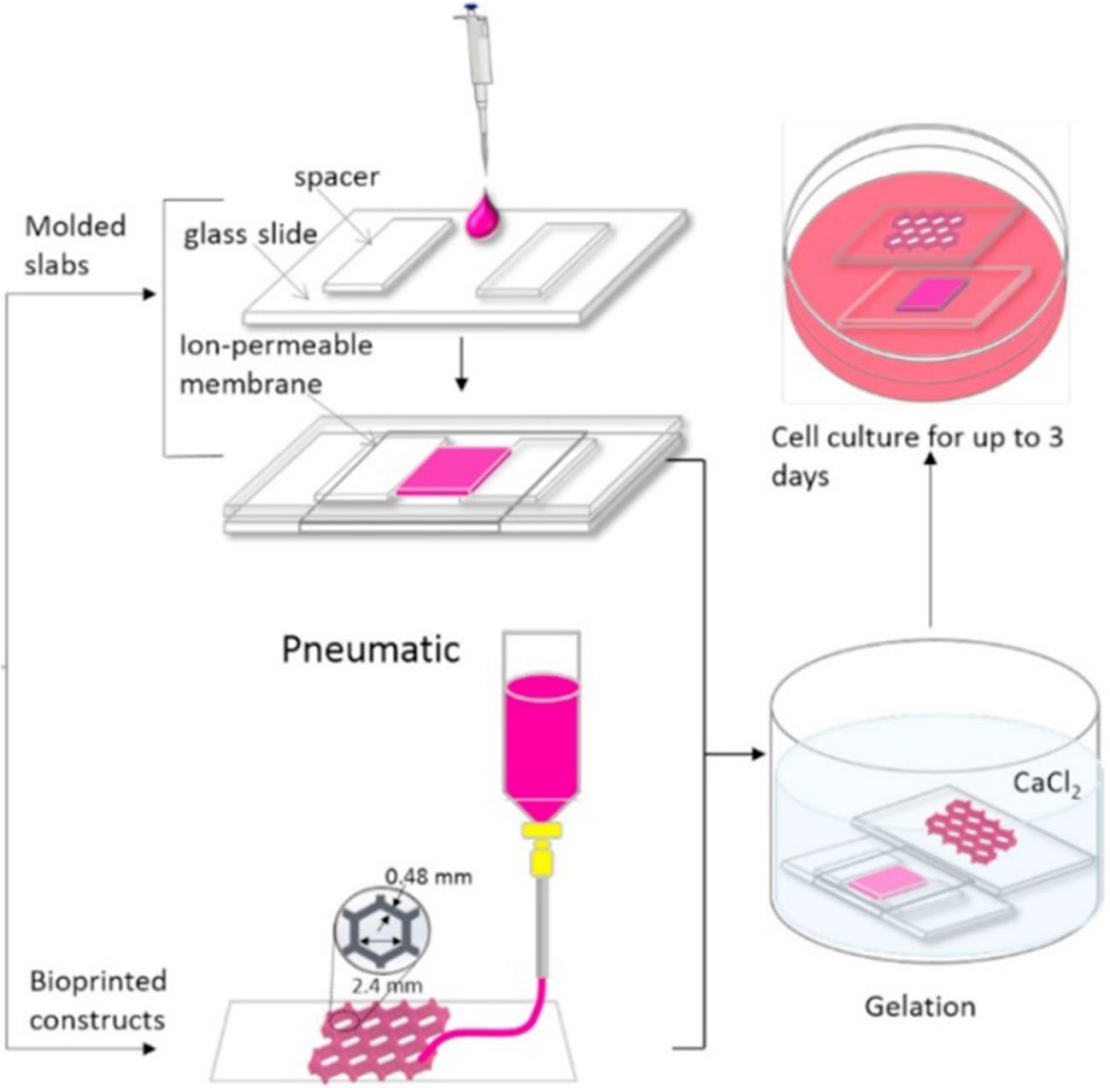
Scheme of hydrogel structure biofabrication [46]

The research of Wu Y. et al. (Wu 2018) related to the 3D bioprinted liver scaffolds using bioink based on sodium alginate, cellulose nanocrystals, fibroblast cells, and liver cells. Hydrogel extrusion technology was employed to obtain the structure. Fibroblasts were used to 3D bioprint the edges of the scaffold, while liver cells formed its central part. The cross-linking agent herein was a solution of calcium chloride. The fabrication procedure is shown in Fig. 8. In particular, the rheological properties of the bioinks (extrusion, shear thinning, and shape fidelity) and the biological activity of the cells were investigated thoroughly. According to the results of biological research, 3D bioprinting had no statistically significant effect on cell viability. The inclusion of cellulose in alginate-based hydrogels improved shear properties and did not affect the viscosity of the bioinks. Furthermore, cells in bioinks did not affect the hydrogel viscosity either. The tests also indicated satisfactory shape fidelity of the hydrogel 3D prints. According to the authors, the best bioink (in terms of 3D bioprinting, structural stability, and cell culture) was an alginate-based hydrogel (2% w/v) with cellulose nanocrystals (4% w/v). As presented here studies were preliminary, the researchers further propose incorporating gelatin into the hydrogel to improve cell binding to its structure [46]. It is worth noting that although the study showed minimal cell damage during the 3D bioprinting process, long-term cell viability and activity were not thoroughly examined. Thus, more extensive investigation is needed.

Other research in this field focused on different hydrogel additives, as presented by Cleymand F. et al. (Cleymand 2021), who developed chitosan-based ink with guar gum biopolymer. The main aims were the fabrication of novel hydrogel ink and the optimization of the 3D printing extrusion process. Enriching chitosan hydrogels with guar gum increased the viscosity of the ink, and simultaneously increased the precision of 3D prints (relative to 3D models). According to the authors, the best hydrogel was based on chitosan (85%) with the addition of guar gum at a concentration of 15%. The hydrogel exhibited improved rheological and strength properties, allowing the AM of three-dimensional structures to be used for potential biomedical applications, e.g., for 3D printing of scaffolds [47]. The study needs to be expanded to assess the biocompatibility and long-term viability of cells within 3D-printed constructs.

Based on the aforementioned articles, critical features of the additive manufacturing of hydrogels were defined and analyzed.

First, the accuracy and resolution of the printing are primarily affected by the material type, thickness of the layer, sterilization procedure, 3D print size, and surface tension.

The thickness of the layer is mainly influenced by the diameter of the nozzle and the surface tension.

The fidelity of the shape is primarily influenced by cross-linking of the polymer ink (polymerization process), the composition of the material, its stiffness, yield stress value, and swelling. The real challenge is to develop a hydrogel that, while maintaining its shape (compatible with the 3D computer-designed model), will exhibit appropriate stiffness without adversely affecting the cell viability.

The biological activity of living cells in the bioink is primarily influenced by, e.g., cell type, cell concentration, residence time, temperature, the porosity of the hydrogel, the formation of shear stresses, viscosity, and viscoelasticity, yield stress value, cross-linking procedure, the biocompatibility of materials or by-products, and polymer concentration.

In turn, the mechanical properties of 3D prints are primarily influenced by cross-linking of the polymer ink, the viscosity of the ink, hydrogel yield strength, temperature, time, cells presence, and their even or uneven distribution, load action on the hydrogel component, layer defects, weak interlayer adhesion, molecular weight of the ink, or amount of liquid inside the hydrogel.

The printability is mainly affected by the viscosity of the biomaterial, surface tension, cross-linking (e.g., cross-linking density), molecular weight, polymer concentrations, shear thinning, biodegradation, biocompatibility, shear rate, or yield stress value.

The viscosity of the ink is primarily influenced by the molecular weight of the material, the concentration of the polymer solution, the complexity of the chemical structure, the temperature, as well as the presence of shear-thinning materials.

The swelling of hydrogels is mainly influenced by cross-linking of the ink, molecular weight, and amount of liquid in the hydrogel (e.g., culture medium).

The degradation and biodegradation rate predominantly depend on several factors including the presence of cells within the hydrogel structure, culturing time, temperature, hydrogel composition, polymer concentration, and the inclusion of hydrophilic and hydrophobic polymers [24].

**Fig. 9:**
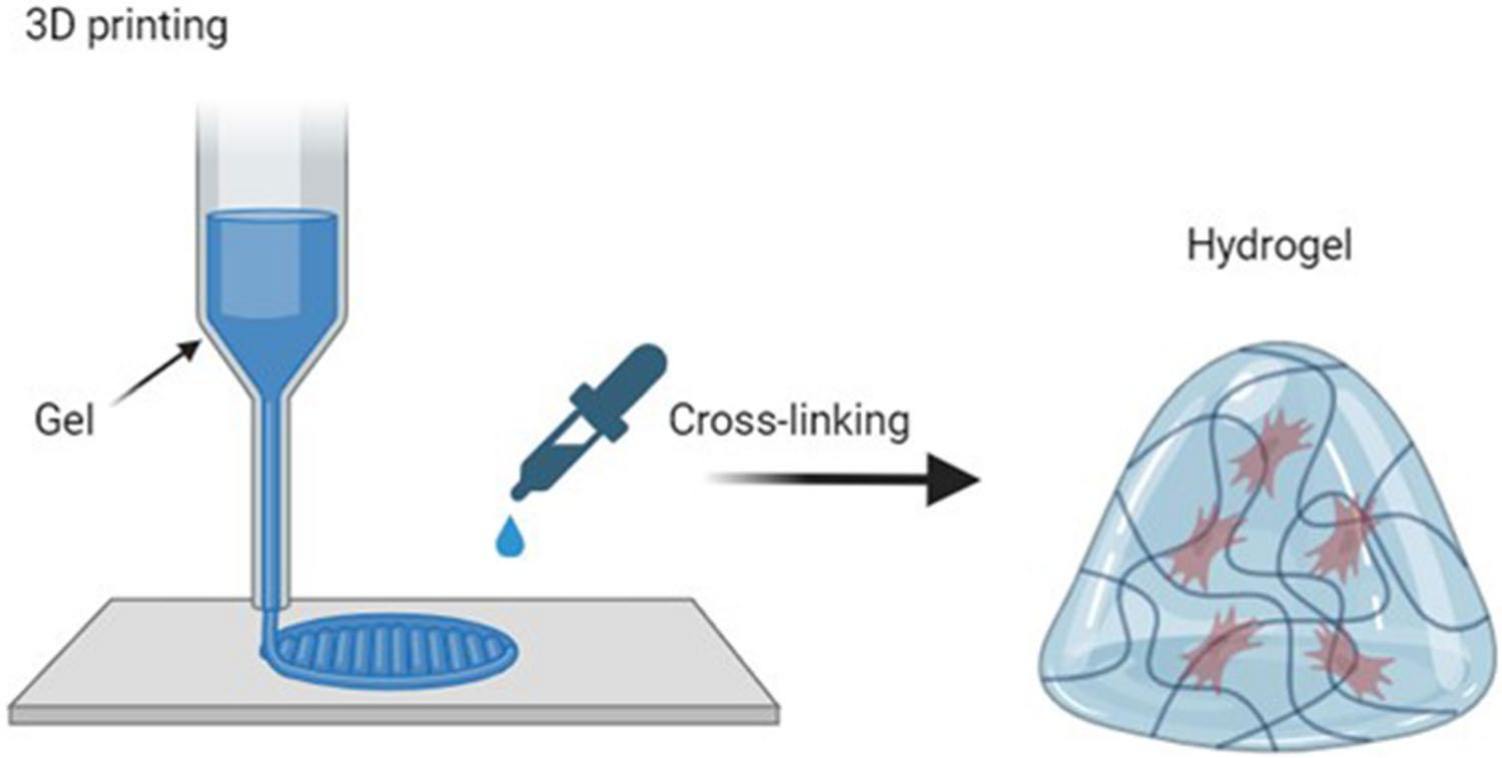
Mechanism of additive manufacturing of hydrogels [73]

Summarizing the issue of hydrogel 3D printing process (Fig. 9), an ideal hydrogel ink should be characterized by the following properties: easy extrusion and printability, the ability to manipulate physical, chemical, and biological properties**,** allowing a homogeneous distribution of cells in its structure**,** biocompatibility (enabling the survival of cells, promoting their biological activity, such as proliferation or migration), required rheological properties, such as adjusted viscosity and stability during printing, as well as after the process [13, 17, 20].

The key advantages of using hydrogels for 3D printing in cell engineering are as follows. 3D printing of hydrogels makes it possible to achieve a microenvironment similar to in vivo conditions (ECM-like properties), including complex geometries of the structure. In the area of hydrogel AM, it is possible to 3D bioprint with cells immersed in bioink or 3D print with hydrogel ink and apply cells already into the printed structure. The choice of 3D printing or 3D bioprinting methods depends on the nature of the research being done, it remains a matter of choice for researchers. The second option is more complex and complicated because of the impact of e.g. printing parameters on cell viability. Nevertheless, hydrogels have a high water content, which improves the diffusion of nutrients and oxygen, promoting cell viability and functions. The appropriate hydrogel composition provides biocompatible properties, which means they can be designed to support cellular activity. The 3D-printed hydrogels integrated into lab-on-a-chip platforms offer several performance enhancements over traditional platforms (e.g., fabricated using glass or PDMS). Additively manufactured hydrogel platforms allow the integration of multiple cell types and the incorporation of microsensors, providing real-time monitoring and feedback within a single microchip. By providing a more accurate representation of the human cellular environment, 3D-printed hydrogel lab-on-chip systems reduce the reliance on animal models, which could allow the elimination of animal experiments in the future. Overall, 3D printing of hydrogel LOC platforms represents significant advances in cell engineering, offering a more accurate, scalable, and ethically sound approach to biomedical research [48, 49].

## Lab-on-chip assisted by hydrogels in cell engineering

**Fig. 10:**
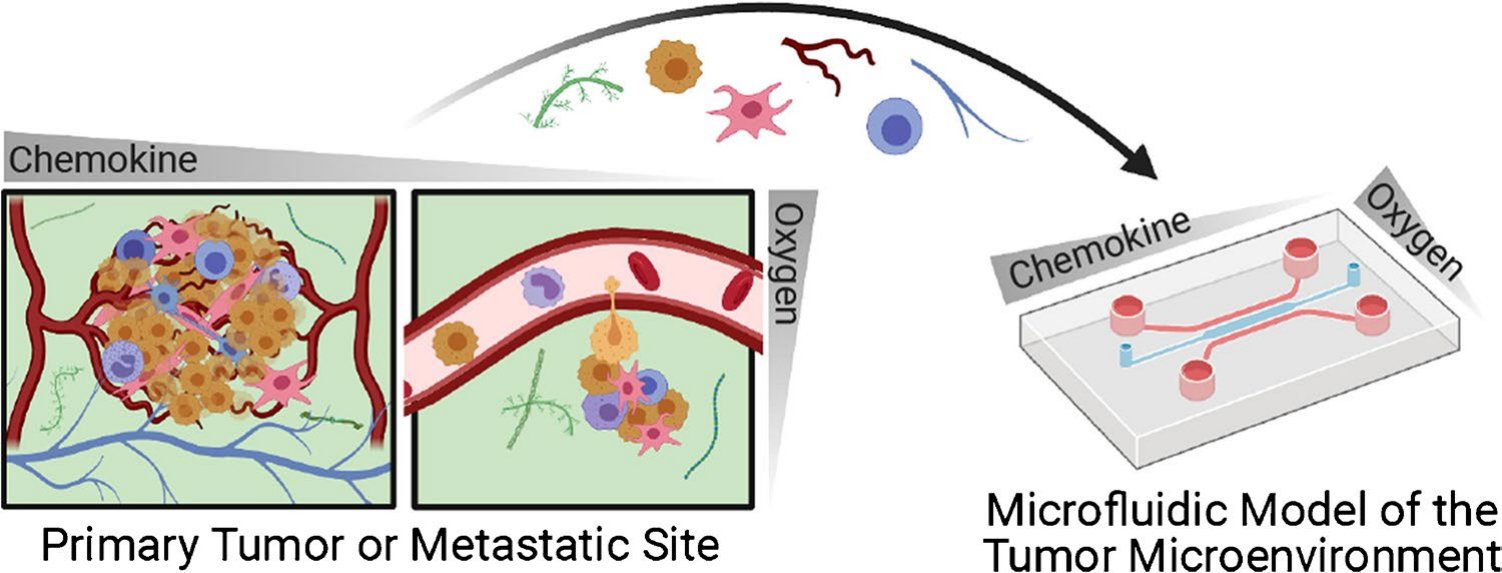
Visualization of the similarity of cancer environments and lab-on-chip platform [74]

Lab-on-chips assisted by hydrogels are innovative solutions for cell cultures in a microscale. Biocompatible hydrogels imitate cell environments, such as the extracellular matrix (ECM), where cells grow, differentiate, proliferate, and migrate. Hydrogels can be treated as cell transporters or as a structural part of the LOC device [24, 50–52]. A representation of the cellular environment in a fluidic microdevice is shown in Fig. 10.

The connection between LOC platforms and hydrogels is described for instance, by Ayuso J. et al. (Ayuso 2016) who developed and characterized a microfluidic device that imitates a tumor environment. The cultivated HCT-116 colon cancer and U-251 MG glioblastoma multiforme cell lines mixed with collagen hydrogel were injected into LOC. First, the authors analyzed the fluid flow through the microchannels that imitate blood vessels. Parallel flow to the central microchamber was observed after injection of 10 µl of the mixture. Thus, there was no interaction with the hydrogel caused by the high specific resistance of collagen filling in the main chamber. In the next step, 300 µl of the medium was supplied through the microchannel and a clear intrahydrogel flow was observed. The authors decided that 1–2 ml of liquid is needed to replace or supplement the cell medium, considering the volume of the central microchamber – 2 µl. Scientists also analyzed cell viability. When the cell concentration applied to the LOC was the highest (40 million cells/ml), many dead cells were found in the central part of the microchamber. Moreover, a band of dead cells formed the so-called necrotic core. However, after reducing the concentration of cells in the hydrogel to 4 or 10 cells/ml, the necrotic core was much smaller. As observed, the dead band of HCT-116 was significantly greater than for MG U-251. Meanwhile, the authors also analyzed cell proliferation and the results showed that cells growing near the microchannels proliferated much more frequently than cells located around the necrotic core. According to this, as the cells moved farther away from the lateral microchannels, the intensity of proliferation decreased until the band of dead cells. What is more important, a similar phenomenon occurs in the tumor environment. The next step was to analyze the oxygen and glucose gradient in the microdevices. The results showed that hypoxia is generated in microdevices but simultaneously, there is a possibility to monitor and evaluate this adverse phenomenon in real-time. Moreover, hypoxia levels can be manipulated by intrahydrogel fluid flow. In the case of the glucose analysis, based on the confocal images, it was found that synthetic glucose rapidly diffused through the hydrogel in the absence of the cells. The difference in fluorescence intensity between the glucose-perfused microchannel and the opposite channel was about 30%. In the presence of U-251 MG cells, the glucose diffusion profile was similar to the LOC without cells. On the contrary, a different glucose gradient was generated with HCT-116 cells, in which a higher glucose fluorescence intensity was observed near the lateral microchannel of the perfused glucose. The gradient increased by about 50% compared to experiments with control or U-251 MG cells. The researchers also assessed quantitively reactive oxygen forms (ROS) to be quite low. The highest amount of ROS was observed near the microchannels, where oxygenation was the largest. The last research concerned drug penetration and its impact on the microenvironment (doxorubicin (DOC)—HCT-116, alkylating agent temozolomide (TMZ)—U-251 MG)). As a result of the experiments, the DOC drug initiated cell death at a high level, mostly near the microchannel. However, this destructive impact was significantly weaker as the necrotic band approached. Referring to the TMZ, a considerably lower cell-killing influence than in the control group (2D cell culture) could be noticed utilizing the LOC platform. Nevertheless, the authors claim that the described and tested LOC platform is suitable for the validation of cancer cell treatments and the development of new cancer therapies using cytotoxic drugs, and further research will be conducted [53]. Nevertheless, this particular model may not fully replicate the complexity of the tumor microenvironment, including the variety of cell types, extracellular matrix components, and signaling pathways present in vivo. The model is primarily designed for short-term experiments, thus there is a need for long-term investigations.

Only a year later (2017), a team headed by Ayuso J. presented research about glioblastoma multiforme on a microfluidic chip, which was fabricated using the photolithography technique. The LOC platform consisted of a central microchamber for the location of cells mixed with collagen hydrogel and lateral microchannels with cell medium. C-6 glioma cells and U-251-MG multiform glioma cells were cultivated. The results of the viability test showed that cell viability in LOC was similar to the results of the control group, exceeding 95%. Moreover, constant fluid flow nourishing the culture did not mechanically affect the hydrogel or embedded cells. The scientists, similarly as in the previous studies, also analyzed the glucose gradient and compared the diffusion profiles between LOC with and without cells. The analysis indicated that it was similar for both cases. On that basis, the potential application of the described solution can be focused on, e.g., drug delivery and drug screening [54]. It is worth remembering that microfluidic chips, despite their many advantages, have limitations in mimicking the full tumor microenvironment. For example, they might not completely replicate the heterogeneity of cell types, ECM components, or the complex interactions between tumor cells and surrounding tissues. Moreover, it is crucial to ensure that observations in the microchip correlate with actual glioblastoma behavior in patients, so there is a need for in vivo studies.

There is also a possibility that hydrogels can be structural parts of a microfluidic device. An interesting literature review in this area was done by Goy C. et al. in 2019 (Goy 2019) [18]. In this paper, e.g., the research of Sun H. et al. (Sun 2016), who focused on the development of a cell-on-hydrogel platform for fast, cheap, and multifunctional research, was described. The microdevice was fabricated by molding method using a polymer solution and casting to a PDMS template. Next, a cross-linking process was provided to receive hydrogel matrix bonded to biocompatible glass substrates. The hydrogel matrix consisted of natural polymers such as agar (1,5%) and sodium alginate (1,5%). What is more interesting, scientists provided *E. coli* bacteria culture on the LOC surface, not in the microchambers or microchannels, to eliminate shear flow. Moreover, diffusion studies using fluorescence microscopy and, among others, fluorescein (332 g/mol) as a deflating substance (its molecular weight is close to the molecular weight of well-known drugs) were conducted. The results showed that there was a constant gradient and an appropriate linearity between the two microchannels. Moreover, it was found that the proposed method led to the generation of a stable gradient for a long time. It was possible thanks to the continuous refreshing of the gradient source and drains by the flow inside the channels [55]. Agar and alginate hydrogels used in these studies are cost-effective and accessible materials, but simultaneously, these hydrogels might have limitations in terms of their mechanical properties, nutrient diffusion, and compatibility with various cell types. The study did not fully address how these factors affect the performance and accuracy of antimicrobial susceptibility testing.

**Fig. 11:**
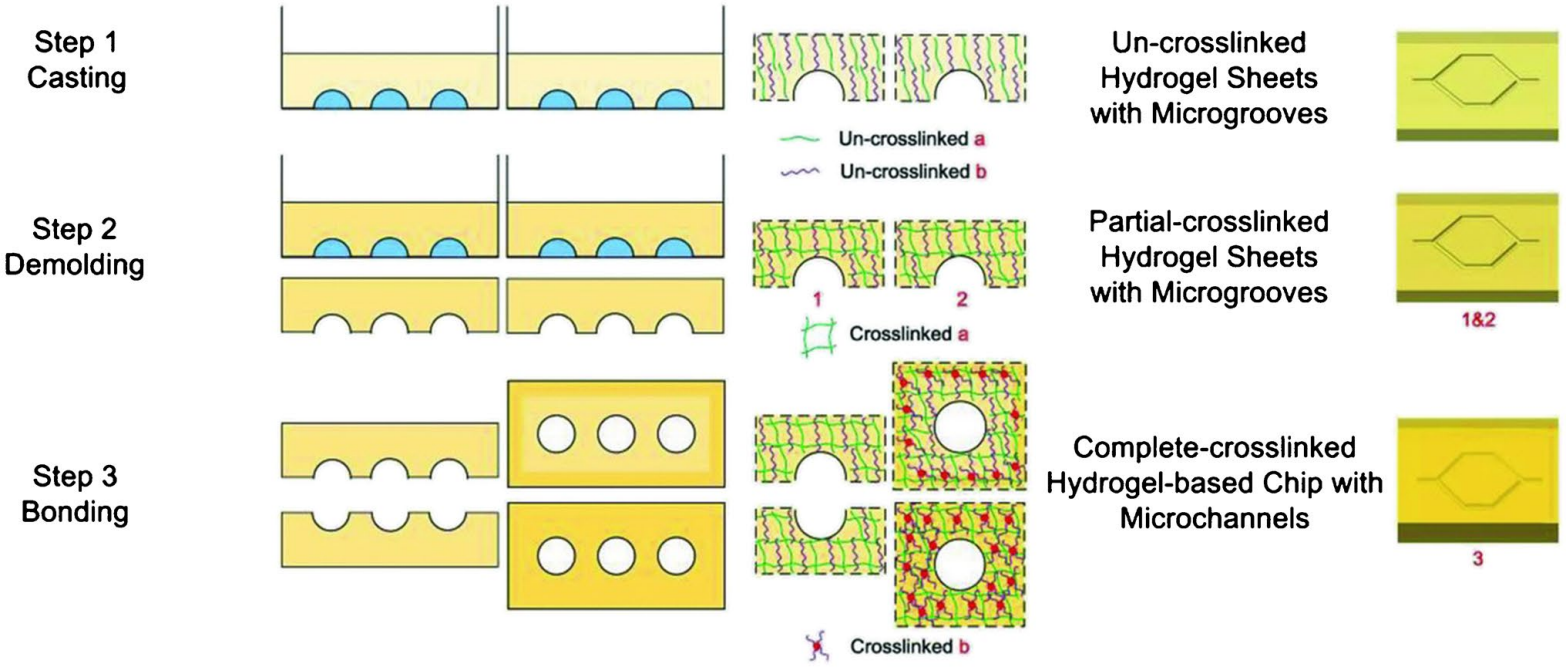
The procedure employed for the fabrication of a microfluidic platform [56]

On the other hand, Nie J. et al. (Nie 2018) investigated vessel-on-chip, based on hydrogels. The scientists used the casting and bonding method to prepare hydrogel structures as shown in Fig. 11. The crucial in this method was a double cross-linking process to obtain an entirely homogeneous structure. There were three hydrogel compositions: alginate and gelatine, alginate and methacrylate gelatin (GelMA), and gelatine and GelMA. In the case of alginate-gelatin hydrogel, the removal process from the mold was based on the gelation properties of gelatin at 4 °C. On the other hand, the binding process was based on ionic cross-linking of alginate with a calcium chloride solution (4% w/v). For alginate and GelMA hydrogel, in turn, the removal process from the mold was based on ionic cross-linking of alginate with Ca^2+^, while the bonding of hydrogel layers was based on photo cross-linking (UV) of GelMA polymer. The last polymer composite, gelatin and GelMA, based on gelatin gelation at low temperature (4 °C) during the mold removal process. The layer-bonding process requires UV light to act on GelMA. The molds for casting polymer solutions were designed in several ways, differing in the geometry of the microchannels. Next, diverse structure verification tests were provided. According to the results, hydrogels based on alginate and gelatin, as well as gelatin and GelMA hydrogels, were characterized by microchannels with high transparency. On the contrary, the third composite hydrogel was structurally imprecise, damaged, and non-transparent. For bioassays, human umbilical vein endothelial cells HUVEC were used and placed on the surface of hydrogel microchannels. All fabricated hydrogels were characterized by an ordered and porous surface, which positively affects the adhesion, proliferation, and migration of cultured HUVEC cells. Based on the Authors’ test results (biocompatibility, microstructure, and processing), gelatin and GelMA composition was chosen as the best. The selected hydrogel allowed for the best representation of vascular function for the biological cells under study [56]. While the vessel-on-a-chip model aims to mimic vascular behavior, the complexity of real blood vessels, including their interactions with various cell types and biochemical signals, might not be fully captured. The simplifications made for practical reasons could limit the model's ability to accurately reflect complex vascular dynamics.

Similar research was performed by He J. et al. (He 2016), who fabricated a gelatine-based hydrogel, but more complex microchannels were proposed. The researchers again took advantage of the gelatine gelling properties at low temperatures (4 °C) and the high degree of biocompatibility of this biopolymer. Cross-linking of the polymer solution occurred by enzyme action. In this study, human umbilical cord endothelial cells HUVEC were cultured in the microchannels. The barrier function of the endothelial microfluidic network was characterized during this research. According to the results, HUVEC cells were able to form a uniform biological layer around the microchannels creating a barrier [57]. Further research in this area could encompass vascularization tests, organ-on-chip fabrication, or drug testing. Nevertheless, it is important to focus primarily on long-term studies of cell cultures and biological activity, and optimization of flows in microchannels to get as close to physiological conditions as possible.

**Fig. 12:**
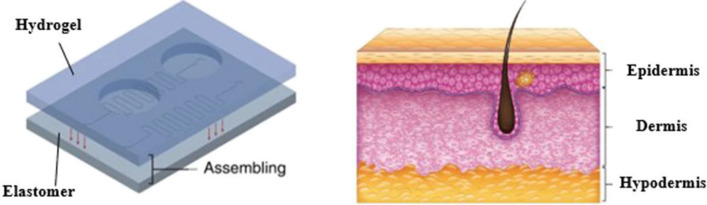
Comparison of the structure of the fabricated lab-on-chip platform versus that of the mammalian skin [58, 75]

Another study was performed by Yuk H. et al. (Yuk 2016) who developed and fabricated a skin-like microfluidic platform based on hydrogel and elastomer films. The crucial aspect herein was to focus on the adhesion of these two biomaterial layers and the structural stability of the designed microplatform. Well-fitted biomaterials, such as elastomers, may be equivalent to the epidermis, while hydrogels might be counterparts of the dermis, as shown in Fig. 12.

The general mechanism for fabricating such microdevice involved pre-polymerization of the hydrogels for geometric stabilization, functionalization of the elastomer surfaces with benzophenone to buffer the effect of oxygen inhibition, and activation of the elastomer surfaces to graft the hydrogel polymer. The last step involved overlapping the two films by cross-linking using UV radiation of the hydrogels overlaid on the elastomeric structures. According to the Authors, the method is versatile for elastomers, such as Sylgard 184, latex, and Ecoflex, and for hard hydrogels, such as polyacrylamide-alginate, polyacrylamide-chitosan, polyacrylamide-hyaluronan, or their equivalents. The manufactured hybrid platforms showed high structural stability and flexibility. According to the mechanical test results, even greater adhesion was indicated by the hydrogel-elastomer combination than by the epidermis and dermis. Additionally, the designed microchannels imitated the blood and lymphatic vessels of mammalian skin. A potential application outlined by the authors is the use of such hybrid platforms to fabricate flexible bioelectronics, i.e., wearables. Moreover, the researchers directed future research toward the additive manufacturing of elastomeric and hydrogel components of microfluidic platforms [58]. Concluding this work, it is worth to notice that the mechanical properties of hydrogel–elastomer hybrids need to be carefully balanced. Hydrogels and elastomers have different mechanical characteristics, and ensuring that the hybrid material maintains desirable properties such as flexibility, strength, and durability under various conditions is crucial. Furthermore, the study showcased potential applications, but the performance of these hybrids may vary depending on the specific use case. That is why there may be a need for additional functionality to optimize the solution.

**Fig. 13:**
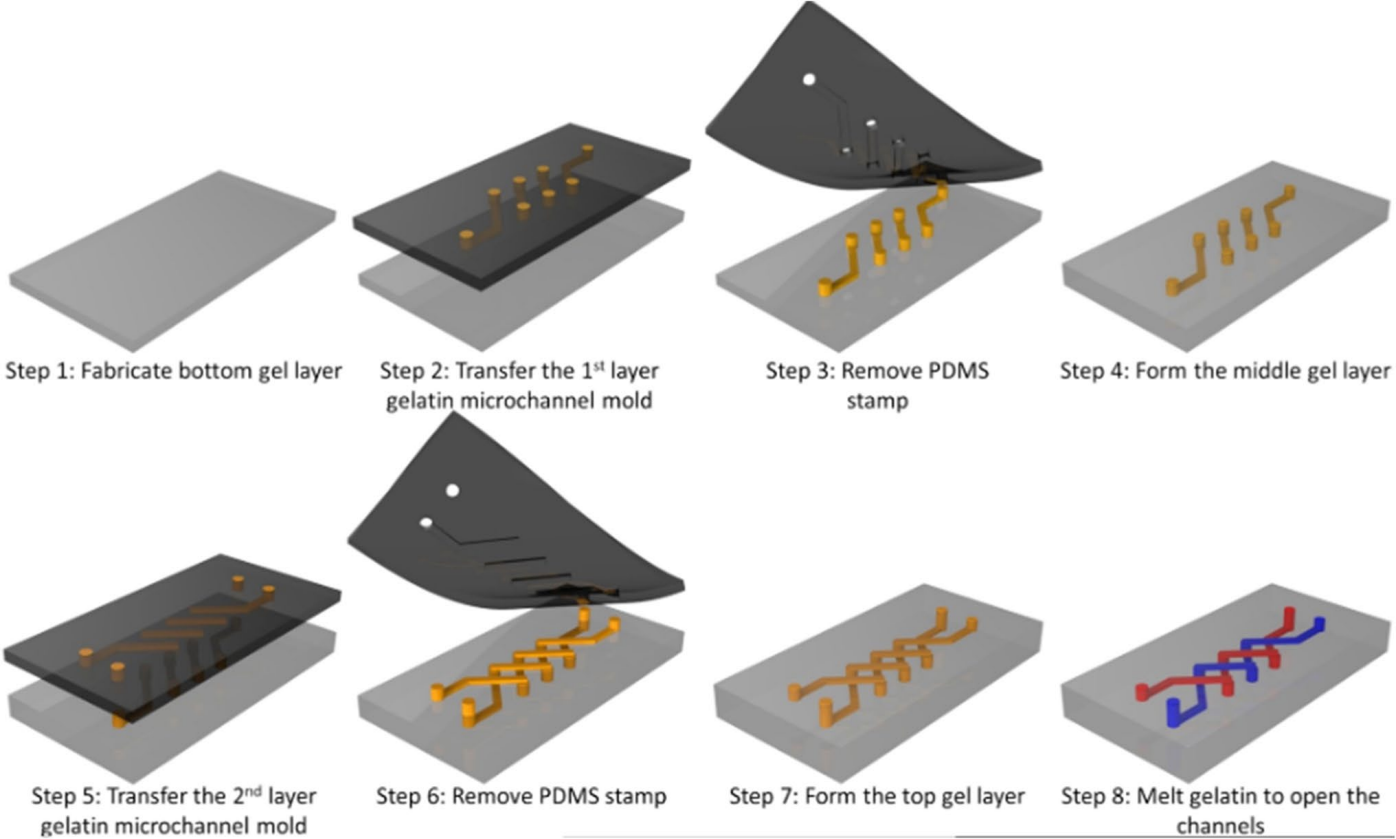
The procedure employed for the fabrication of the multilayer microfluidic platform [59]

Microfluidic platforms, based on silk hydrogel, were also recently developed by Zhao S. et al. (Zhao 2016). Silk is a biocompatible protein material with controlled degradation, which can be suitable for in vivo applications. The process of fabrication of the multilayer microfluidic silk hydrogel using gelatin molding and the layer-by-layer method is presented in Fig. 13. To obtain microchannels, gelatin was melted at 37 °C. To provide appropriate durability of the hydrogel platform, it was encased using PDMS and acrylic. Next, structure performance was verified and according to the mechanical test results, the stiffness of the hydrogels could be adjusted in the range of 1 kPa to 1 MPa, which corresponds to the values of natural biological tissues, demonstrating the feasibility of forging the presented hydrogel biomaterial. However, different concentrations of the silk solution and the content of the crystalline β-sheet affected the stiffness of the structure (the higher the concentration, the higher the stiffness of the hydrogel). As mentioned in the text, the major advantages of the structure were, e.g., high transparency (essential for in vitro research), stability, and biocompatibility. Thus, biological research could be performed utilizing the solution. Human HUVEC cells were cultured in chip microchannels. In turn, human fibroblast cells were cultured within the hydrogel bulk. In both cases, long-term cultures with a high degree of viability and proliferation were obtained. Moreover, in contrast to microfluidic solutions based on PDMS, it was possible to confluently culture HUVEC cells in microchannels (the cells migrated and aggregated to form a whole). In the next research, the authors will focus on the optimization of fabrication methods, which could reduce the process time and labor intensity [59]. Possibly, additive manufacturing of hydrogel layers might be applied for this purpose. Nevertheless, it should be highlighted that silk hydrogels can exhibit variability in their mechanical and biochemical properties depending on their preparation methods and the source of the silk.

**Fig. 14:**
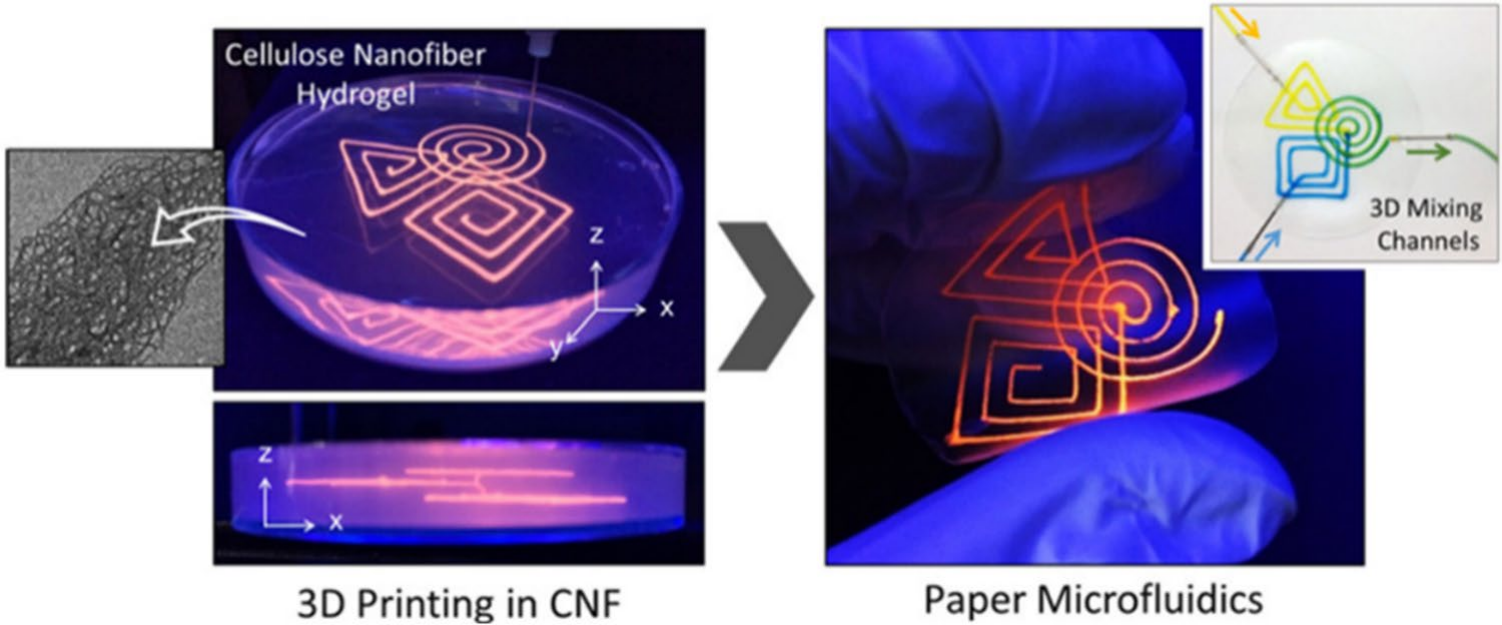
Flexible microfluidic thin film with multilayer microchannels manufactured by 3D printing on a nanocellulose hydrogel matrix [60]

On the other hand, Shin S. and Hyuna J. (Shin 2017) developed and additively manufactured a hydrogel matrix based on cellulose nanofibers as a paper microfluidic device. The fabrication process was the 3D printing of a structure utilizing a mixture of vaseline and paraffin in a nanocellulose hydrogel mass (0.4–1.0% w/v). The following step was drying the print (lining a flexible film) and successively removing the vaseline-paraffin ink by raising the temperature above 70 °C with air assistance. According to the paper, a versatile approach was shown, and it was possible to manufacture different geometries of microchannels (Fig. 14). The microfluidic structure showed high transparency, stability, and excellent rheological properties, which can be used for the fabrication of a wide range paper-based biosensors [60]. Analyzing the article, the mechanical strength and durability of microfluidic devices based on cellulose nanofibers are critical to their practical applications. Research should be expanded to include the effects of mechanical stress and the long-term stability of microfluidic devices.

**Fig. 15:**
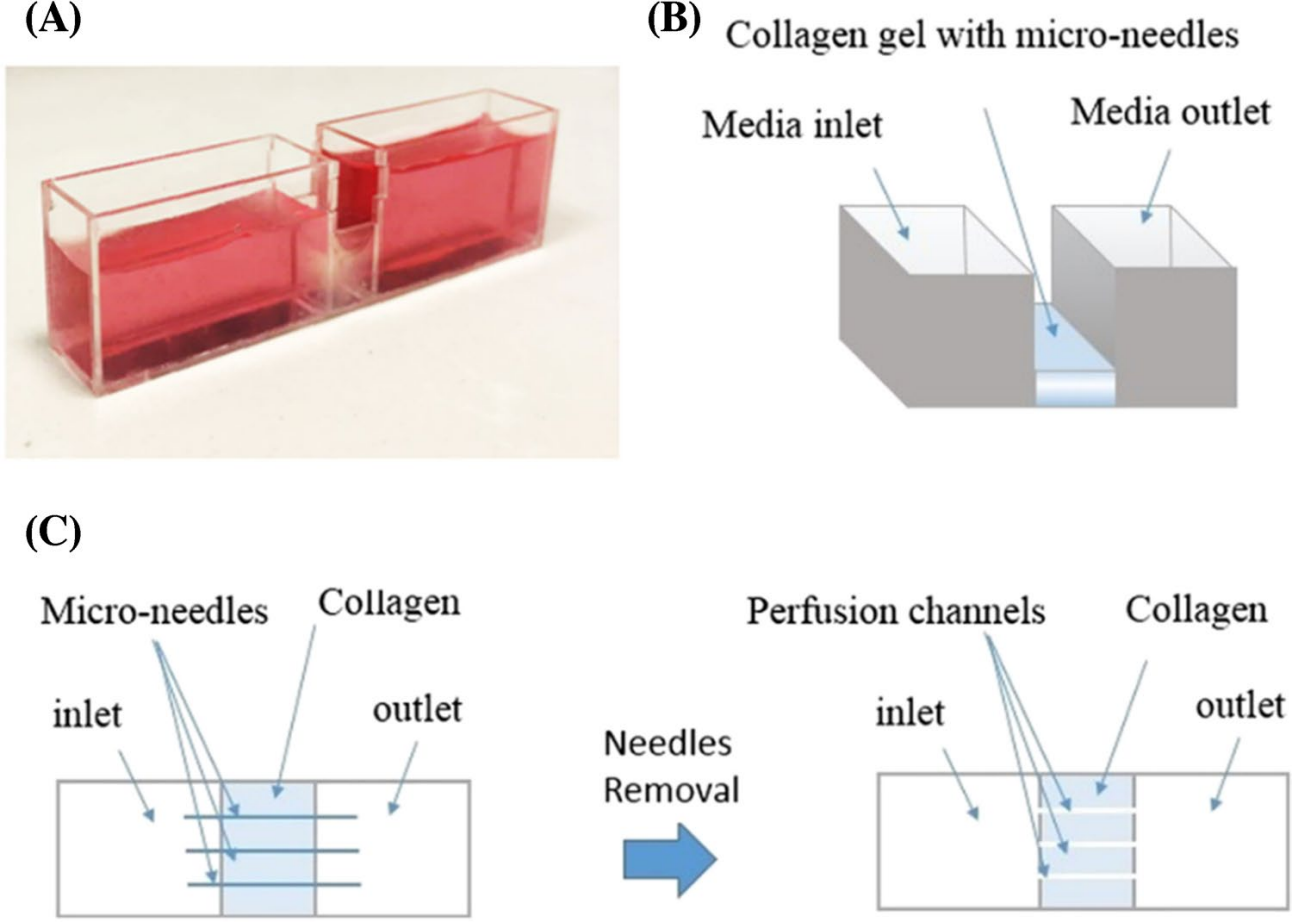
Scheme of microfluidic device and fabricated prototype. **a**) PMMA device and collagen gel in the middle chamber and culture media inside the tanks, **b**) scheme of the microfluidic solution, and **c**) scheme of microfluidic device fabrication [61]

One year later, the team of Jafarkhani M. et al. (Jafakhani 2018) proposed a procedure to optimize additive manufacturing of microfluidic hydrogel structures. The goal was to achieve the best geometry and size of the microchannels for cell cultures and the transport of nutrients and by-products. The collagen-based hydrogel (1, 2, 3% w/v) was prepared using the molding technique. The microchannels were implemented by AM using microneedles (diameters: 100, 300, 500 μm) removed after the collagen hydrogel solidified, as shown in Fig. 15. The scientists performed physical–chemical and biological studies on the prepared hydrogel LOC. In this research, commonly known human umbilical vein endothelial cells (HUVEC) were used. The studies showed, among others, that as the concentration of collagen increased, the porosity of the hydrogels decreased and thus, the efficiency of diffusion and transport of substances also decreased. Regarding cell behavior, more efficient transport of culture medium through the microchannels increased HUVEC viability. Authors claim that the platform will be used in further studies to evaluate its practical application for regenerative medicine and tissue engineering [61]. The authors should focus future research, among others, on the long-term stability and durability of the structure.

**Fig. 16:**
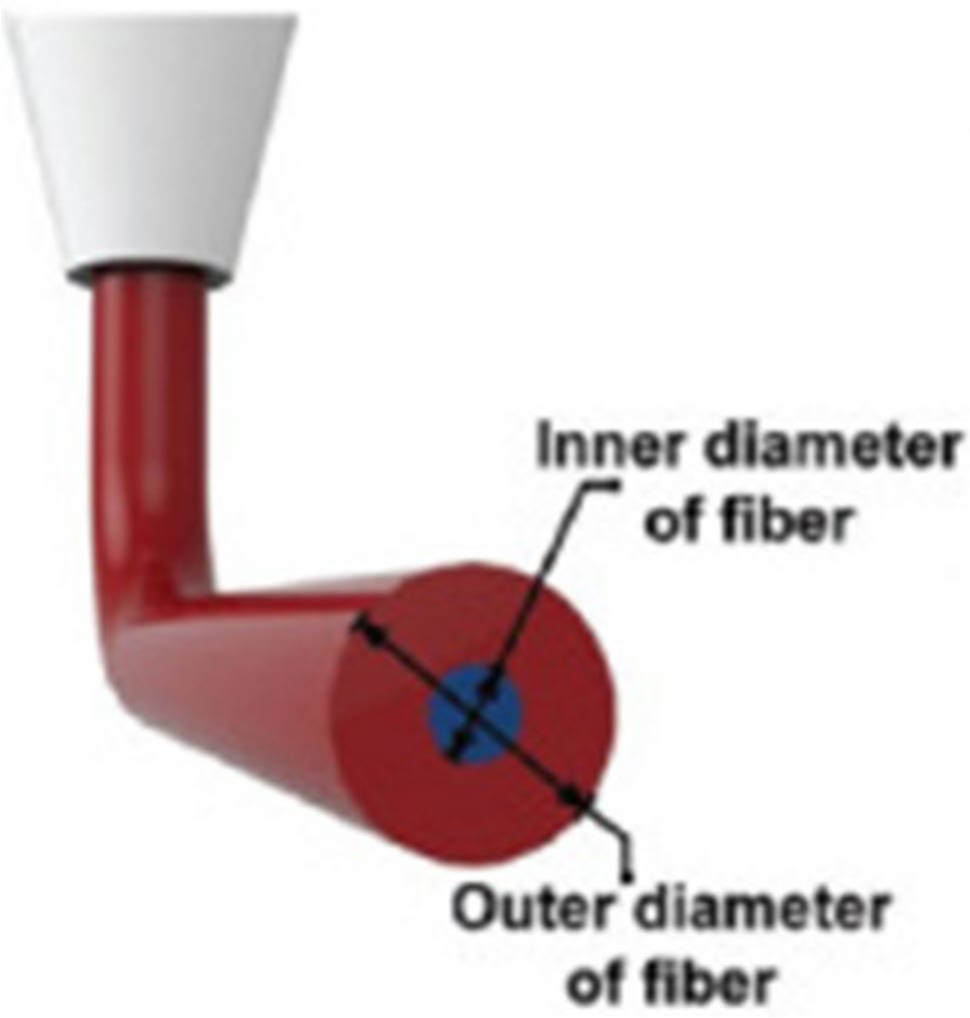
Schematic picture of outer and inner diameters of the fiber [62]

A significant application of additive manufacturing of LOCs is the fabrication of perfusion structures using 3D bioprinting techniques. An example is a micro perfusion device for antiangiogenic drug screening—a type of vessel on a chip, developed by Gu Z. et al. (Gu 2022). The aims of the research were the long-term perfusion and real-time monitoring of angiogenesis (the formation of new blood vessels). The authors focused on biaxial manufacturing by extrusion (Fig. 16) using hydrogels consisting of well-known GelMA (5% w/v) and gelatin (5% w/v) polymers. To prepare the bioink, human umbilical vein endothelial HUVEC cells (3 × 10^6^ cells/ml) were applied. Key elements of this research included the use of a polycaprolactone (PCL) stent, which provided structural support to the tubular lumen, ensuring consistent perfusion over extended periods. The cover of the LOC was manufactured using polyester, PLA, and PDMS components. The authors compared the hydrogel constructs with and without a PCL stent. According to the mechanical test results, at the same stress, higher strength was demonstrated by the samples with a stent, exhibiting lower deformations (by about 10%). Consequently, samples consisting only of hydrogel were more likely to collapse. Biological experiments showed clear differences in cell growth at different drug concentrations, highlighting the great potential of the system for precision drug screening. The ability to maintain perfusion for up to 10 days, depending on the hydrogel used, underscored the robustness of the system and its suitability for long-term studies. Traditional methods of testing these drugs often fall short in replicating the complex, three-dimensional (3D) environment of human tissues. The vessel-on-a-chip system addresses these limitations by offering a more physiologically relevant platform for drug testing. This vessel-on-a-chip technology not only advanced antiangiogenic drug screening but also held promise for broader applications in tissue engineering, pharmacokinetics, and regenerative medicine. By providing a more accurate and controlled environment to study vascular dynamics, this system could significantly improve the development and testing of new therapeutic agents [62]. From the critical point of view, despite its advancements, the study acknowledges certain limitations. The complexity of replicating the full range of physiological conditions in vitro remains a challenge. Additionally, the long-term stability and scalability of the model for high-throughput screening require further exploration.

**Fig. 17:**
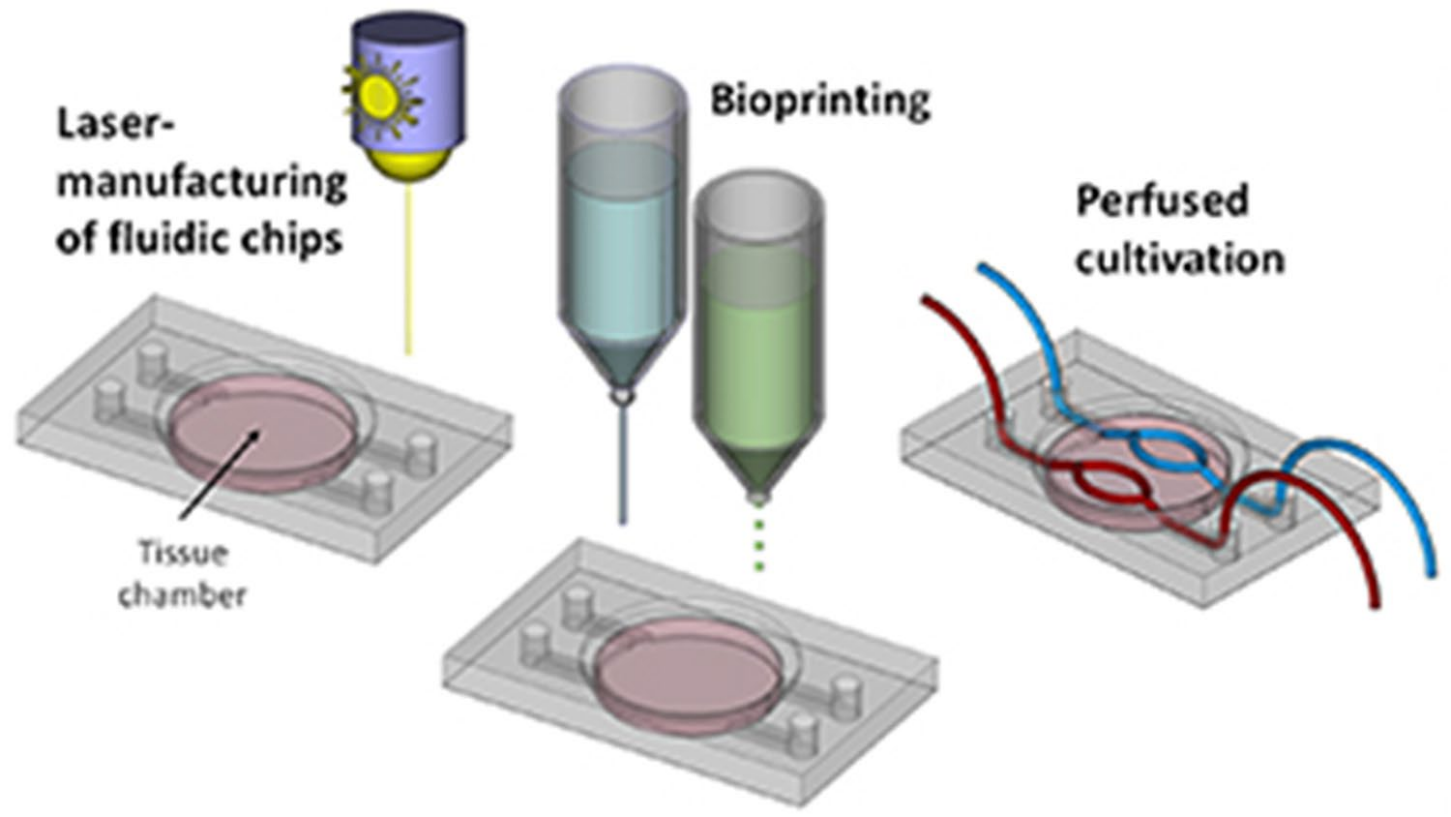
Scheme of 3D bioprinting of soft tissues into laser-engraved microfluidic chips [63]

Another approach is described by Nothdurfter D. et al. (Nothdurfter 2022), who 3D bioprinted vascularized tumor environment in microfluidic chip devices for drug testing, as shown in Fig. 17. Scientists made efforts to mimic the complex tumor environment. Traditional two-dimensional cell cultures and animal models are often unable to accurately replicate the human tumor microenvironment, preventing the development of cancer therapies for this malignant childhood disease. The authors focused their study on examining tumor growth, metastasis, and drug response. The following cell lines were used in the study: neuroblastoma cells and endothelial cells, which are essential for the formation of the vascular network within the tumor. The microchannels in the printed tumor were prepared through the use of Pluronic—F127, a commercial hydrogel that dissolves at 15 °C. The LOC platform consisted of interconnected layers of polymethylmethacrylate (PMMA). The microfluidic device allowed researchers to study the effects of different concentrations and combinations of drugs in real-time. This approach may lead to more accurate predictions of how a patient’s tumor would respond to treatment, allowing for the development of more effective, tailored therapies in the future. This points to high developments in personalized medicine. An important innovation in this study is the successful incorporation of the vascular network into a 3D bioprinted tumor model. Vascularization is crucial because it facilitates the delivery of nutrients and oxygen to tumor cells, mirroring the behavior of real tumors in the human body. This vascularized environment also allows for the study of drug delivery and efficacy under conditions that closely resemble human physiology. The study showed that the presence of a vascular network significantly affects the behavior of tumor cells, including growth patterns and drug resistance [63].

## CNT-doped hydrogels

**Fig. 18:**
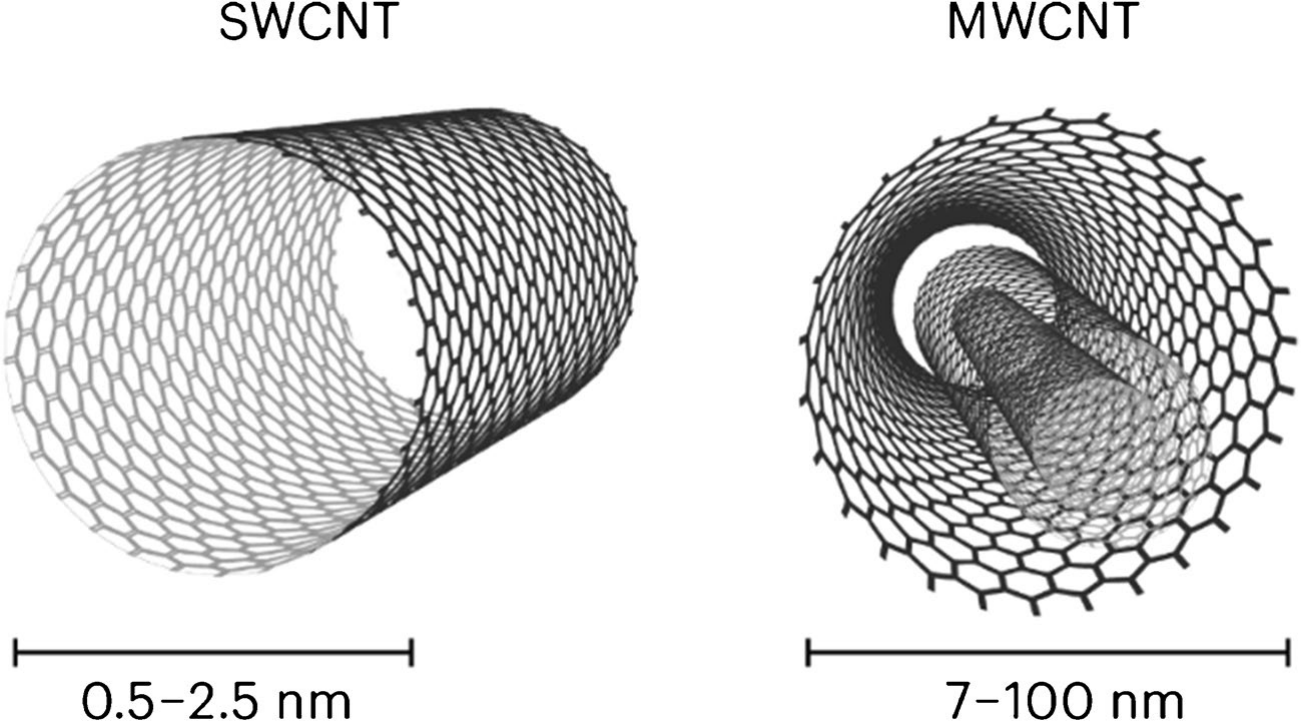
Visualization of two main types of carbon nanotubes, on the left SWCNT, on the right MWCNT [76]

**Fig. 19:**
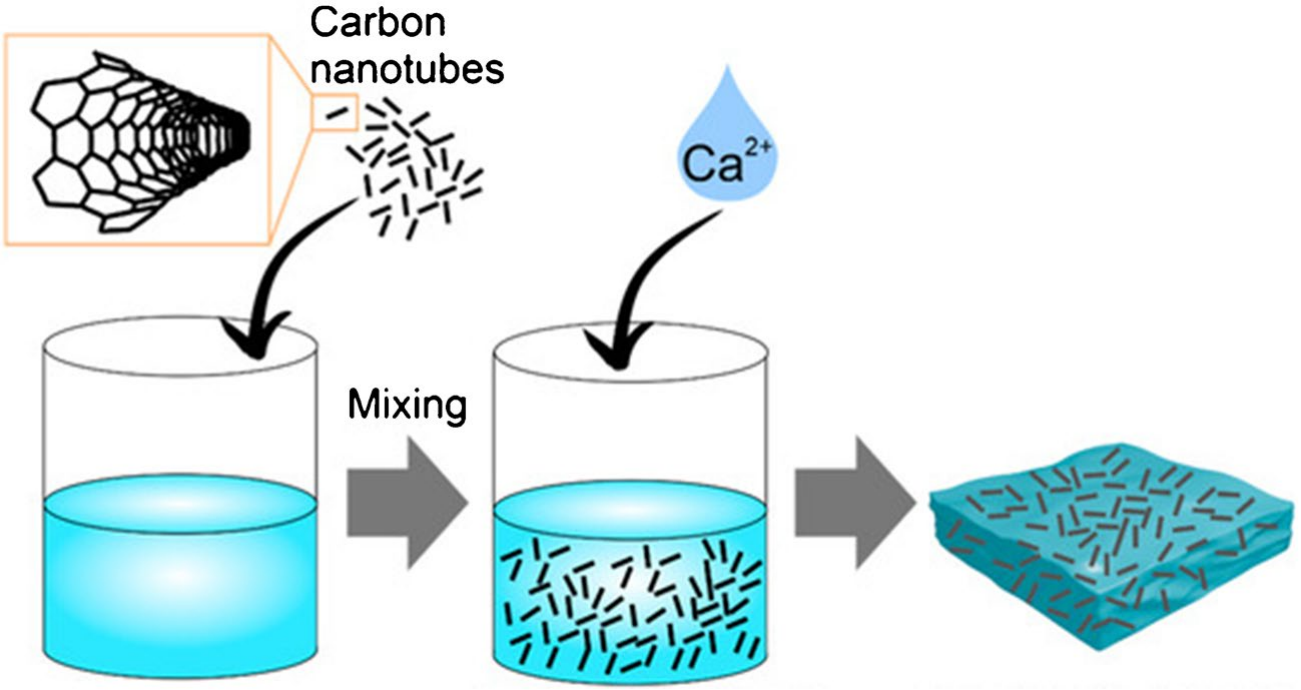
The manufacturing process of hydrogel enriched with carbon nanotubes [14]

Moving on, carbon nanotubes (CNTs) are divided into two main groups, such as SWCNT and MWCNT, as shown in Fig. 18. As mentioned, CNT can mimic human collagen, because of its fibrous form and similar dimensions. Carbon nanotubes are biocompatible and increase biosensor sensibility, e.g., biosensor-on-chip (BOC) [19, 64]. CNTs can be added to hydrogel structure based on collagen, gelatin, alginate, chitosan, or agarose, to enhance its performance in different biomedical solutions. The combination of hydrogels and CNTs increases the biocompatibility of the material. CNTs also improve mechanical stability, Young modulus, cell adhesion, proliferation, differentiation, and tissue formation. In addition, CNTs reduce hydrogel impedance, which allows for different electrical stimulation studies [64]. The example procedure for CNTs-based hydrogel fabrication is presented in Fig. 19 [14]. The following paragraphs of the chapter describe the articles concerning CNT application in the interesting field of hydrogel LOCs.

For example, Chen X. et al. (Chen 2023) researched 3D printing of graphene oxide (GO)/carbon nanotubes (SWCNT) combined with sodium alginate. The scientists analyzed, among others, the viscosity and mechanical properties of the hydrogels. It was shown that after adding GO and CNT, the viscosity of the structures increased, but only by 12.4%. Thus, it was still within the material viscosity range that can be printed. In turn, to check the mechanical properties of such hybrid structures, scientists performed uniaxial tensile testing of hydrogel samples, and Young’s modulus (longitudinal modulus of elasticity) was calculated. The results showed that Young’s modulus was higher (more than 6.5 times) in the GO and CNT-enriched hydrogels compared to the control hydrogels (pure sodium alginate). In summary, CNT increased mechanical stability and did not affect the 3D printing process since the appropriate viscosity of the ink was ensured [65]. It is noteworthy that the study used as many as two additives that change the functionality of the origin material. As a result, the study is more complicated and complex. Moreover, it can be difficult and not repeatable to achieve an even distribution of carbon additives in the hydrogel matrix, which affects the quality of the final product. In the future, a potential problem may arise in larger-scale production due to the high purchase costs of CNTs or graphene oxide.

Further research by Abalymov A. et al. (Abalymov 2021) focused on gellan gum hydrogel (GG, 1%) enriched with CNT of different concentrations (0, 0.5, 1.0, 1.5%). It was found that the presence of CNT affects hydrogel bulk significantly. For instance, the hydrogel pore size decreased as the CNT concentration increased. Therefore, a more regular structure was presented by hydrogels with higher amounts of CNT. The roughness of the hybrid hydrogel was also higher and comparable over a range of CNT concentrations. The following research focused on mechanical compression tests to indicate the longitudinal modulus of elasticity. The obtained values showed that this parameter was significantly lower for hydrogels with the highest CNT concentration (1.5%) compared to hydrogels with a lower CNT content (0.5, 1.0%). This phenomenon could be explained by the disruption of the structural integrity of polymeric compounds by the incorporation of CNTs into hydrogels. In these studies, some preliminary biological investigation was also conducted to evaluate the biocompatibility of the solution on the example of MC3T3-E1 osteoblast cell cultures. For instance, the best cell adhesion was indicated for hydrogels with the highest CNT concentration. Similarly, the highest cell viability was indicated for 1.5% CNT hydrogels, which is consistent with the relation that low cell adhesion results in cell death. To confirm the presence and distribution of CNT in hydrogels, the scientists performed experiments related to light absorbance and spectroscopy. The results showed that the higher the CNT concentration, the higher the absorbance coefficient (light absorption). In this article, tests concerning hydrogel swelling were also done. Swelling levels were compared between the GG hydrogel and the 1.5% CNT hydrogel. The conclusion was that high concentrations of CNT reduced the degree of hydrogel swelling. In summary, CNT addition in hydrogel matrices can enhance the mechanical properties of hydrogels and simultaneously be beneficial for different cell engineering studies [14]. The authors, in their follow-on research, should focus on the long-term mechanical and chemical stability of CNT-gellan gum composites, which will complete the transferred knowledge on gellan gum hydrogels enriched with CNT for biomedical applications.

In turn, the research of Van den Broeck L. et al. (Van den Broeck 2019) focused on carbon nanotube-reinforced polyethylene glycol composite hydrogels (PEGCNT). CNT was used in different concentrations, such as 0.015, 0.03, 0.045, 0.15, and 0.2%. Moreover, L929 fibroblast cells were cultured in these structures, as in cell growth matrices. As a result of the tests, at first, the hydrogel swelling coefficient was evaluated and was found not to be changed after CNT addition. Therefore, carbon nanotubes did not affect the hydrogel cross-linking density. The rheological investigation of the properties of the biomaterials showed that PEGCNT hydrogels (0.015%) received higher storage modulus values (1915.0 ± 101.7 Pa) than PEG hydrogels (1196.7 ± 125.0 Pa). On that basis, CNT addition notably reinforced the hydrogel structure, without providing substantial chemical changes. Moreover, compression tests were performed on the swollen hydrogel samples and higher values of modulus of elasticity were presented by hydrogels with CNT (PEGCNT). The last research related to biological investigation towards cell viability assessment in contact with the developed hydrogel structures. The tests showed that L929 fibroblasts survived in all prepared biomaterials, and cell viability was higher for PEGCNT hydrogels (83.3 ± 10.7%) than for PEG hydrogels (51.9 ± 8.3%). According to the paper, similar correlations were discovered in the case of nerve cells [66] and cardiomyocytes of rats [67]. Scientists believe that this phenomenon is related to the random distribution of CNTs in the hydrogel matrix, which resembles collagen fibers and ECM properties. In summary, research confirmed enhanced biocompatibility of CNT combined with hydrogels, which was advantageous for cell adhesion, proliferation, and differentiation [64]. Nevertheless, long-term biocompatibility and potential cytotoxic effects of CNTs need to be investigated.

Wang R. et al. (Wang 2019) worked on hydrogel composites consisting of polyelectrolytes enriched with CNT. This research showed, i.e., that the proposed structures had better mechanical properties compared to the pure hydrogel. CNT-enriched hydrogels exhibited, e.g., higher tensile strength. As the concentration of CNTs increased, the strain increased and the fracture stress decreased. Moreover, the CNT-doped hydrogels were characterized by different adsorption properties compared to the control hydrogels. Namely, CNT-doped hydrogels showed greater adsorption of methylene blue. On the contrary, in the case of rhodamine B (a carcinogenic compound), pure hydrogel exhibited better adsorption [68]. Further research in this field, especially in the context of optical hydrogel properties, is needed to unambiguously evaluate hydrogel applications for biomedical studies, in which cell staining is a standard procedure.

Fan X. et al. (Fan 2022) developed a hydrogel consisting of sodium alginate, polyvinyl alcohol, acacia-magnesium tannin, and silver. and carbon nanotubes (CNT content in the hydrogel was 0, 10, 20, and 30 mg). The procedure for preparing the hydrogel is described in detail in the source article [69]. The proposed hydrogel was characterized by extremely high extensibility (717%), biocompatibility, good adhesion, and self-healing properties with an efficiency of more than 99%. According to the microscopic observations, the homogeneous distribution of carbon nanotubes in the hydrogel structure was confirmed. The presence of CNTs improved the electrical conductivity of the hydrogel (approximately two-fold increase in the conductivity of the CNT-doped hydrogel compared to control hydrogels), as well as increased the mechanical stability. Based on the results from the mechanical tests and, more specifically, tensile tests, it was found that as the CNT concentration in the hydrogels increased, the tensile strength increased and the hydrogel strain decreased. Moreover, the structure exhibited antibacterial properties due to the content of silver nanoparticles. The Authors proposed using the designed hydrogels in the fabrication of wearable electronics to continuously monitor human activity at room and low temperatures [69]. In this context, there is a need to perform in vivo tests evaluating the long-term impact of the CNT or Ag nanoparticles on e.g. human skin.

In summary, the biggest challenges for researchers in the use of carbon nanotubes include achieving their uniform dispersion in the hydrogel matrix. CNTs tend to agglomerate due to strong van der Waals interactions, which can result in local concentrations, non-uniform material properties, and reproducibility problems. Others are a matter of cost, as CNTs have a high purchase cost.

## Photodynamic therapy on-chip

Lab-on-chip microfluidic devices can be used for cell culturing and photodynamic therapy applications to further facilitate the validation of new PDT strategies. As mentioned earlier, PDT is a non-invasive alternative to chemotherapy or radiotherapy treatment [16].

**Fig. 20:**
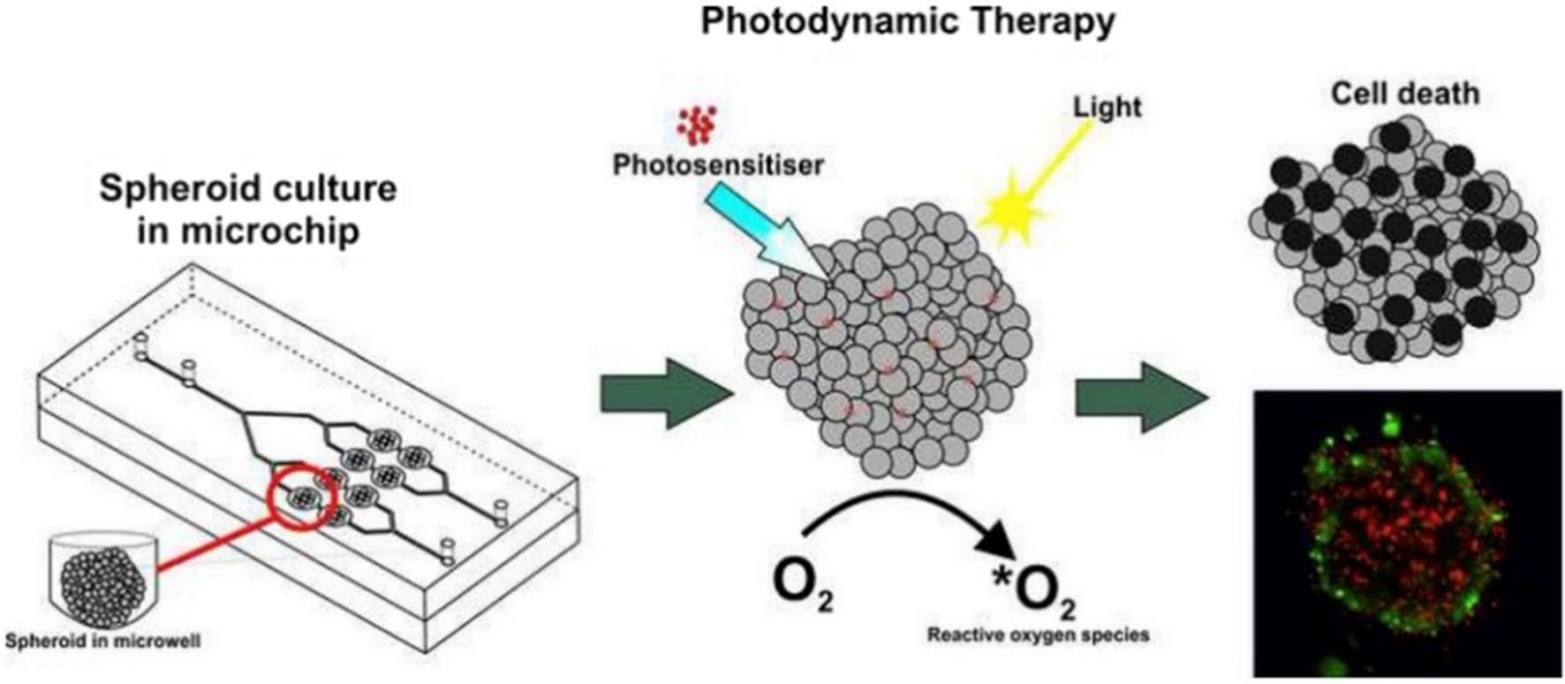
Mechanism of action of photodynamic therapy directly on the chip [16]

In recent times, interesting research on three-dimensional lung spheroids cultured on the lab-on-chip platform for PDT validation was conducted by Żuchowska A. et al. (Zuchowska 2017). The authors fabricated LOCs with microchannels and microchambers using PDMS material and casting method. A549 cancer cells and normal fetal lung fibroblasts MRC-5 were cultured. The PDT procedure employed in this work is presented in Fig. 20. Different concentrations of ALA were used (0.75, 1.00, 2.00, 3.00, 5.00, and 9.00 mM) as a photosensitizer precursor. The light source was a light-emitting diode (LED).

First, the cytotoxicity of ALA was assessed and the studies showed that it did not significantly influence the viability for both cell types (cell viability was about 90%). The next analysis was related to the accumulation of PpIX in the tested cells. The aim was to explain the viability differences between A549 and MRC-5 cells after PDT treatment. As a result, the measured fluorescence intensity was much higher for the first cell type. According to the knowledge, the fluorescence intensity (in quantitative cell viability measurements) is directly proportional to the metabolic activity and viability of the cells. Furthermore, the fluorescence intensity was 1.35 times higher for ALA treatment (3 mM) than in the control group (0 mM ALA). Therefore, it was confirmed that the photosensitizer accumulated selectively in cancer cells was toxic only in the presence of light. Reactive oxygen forms (ROF) were produced during the successful treatment with PDT but only in A549 cells [16].

The team of Jedrych E. et al. (Jedrych 2011) also evaluated PDT treatment utilizing LOC, at which A549 cells were cultured. This time, the microfluidic device was made of PDMS and sodium glass. The scientists found out that PpIX accumulation is related to the ALA photosensitizer concentration. Furthermore, the number of dead cells was comparable to the PDT research performed in Petri dishes. To summarize, cell culture in the proposed microdevice ensured adequate cell adhesion and proliferation. Simultaneously, absorbance measurements reflected the cell accumulation of PpIX. It was found that PpIX accumulation depended on cell type [70].

Another study by Marzioch J. et al. (Marzioch 2015) focused on the observation of cell metabolic processes on the LOC platform during PDT. Lab-on-chip was fabricated out of borosilicate glass. T-47D human breast tumor cells were cultivated within the chip microchambers. Once again, ALA (50, 70, 90 uM) was used as a precursor of the photosensitizer and LED as a light source (lightning time: 0, 2, 5 min.). After preliminary tests, the highest ALA concentration and the longest lightning time were chosen for further research. The authors measured cellular respiration before, during, and after PDT treatment. At the beginning (before PDT), there was a decrease in oxygen concentration due to cell respiration, cell proliferation, and an overall increase in oxygen consumption. After PDT, the oxygen concentration was constant, thus cellular respiration did not occur, which ultimately resulted in cell death. In comparison, control LOC (the same cell cultivation, but without performing photodynamic therapy) was characterized by a continuous decrease in oxygen concentration—the cells consumed oxygen and proliferated. Especially interesting in this research was the repopulation effect observed after the PDT. Treated cells could regenerate, which was confirmed by oxygen consumption. The scientists also measured temperature to check if the light treatment heated up the microdevice and cellular temperature. It was found that the light source did not significantly increase the temperature of the LOC or cultivated cells. Therefore, exposure to LED light has no negative effect on PDT results. To sum up, continuous observations of cell metabolism presented in this paper can be considered as more reliable than simple live/dead assays, which show only final cell viability [71].

**Fig. 21:**
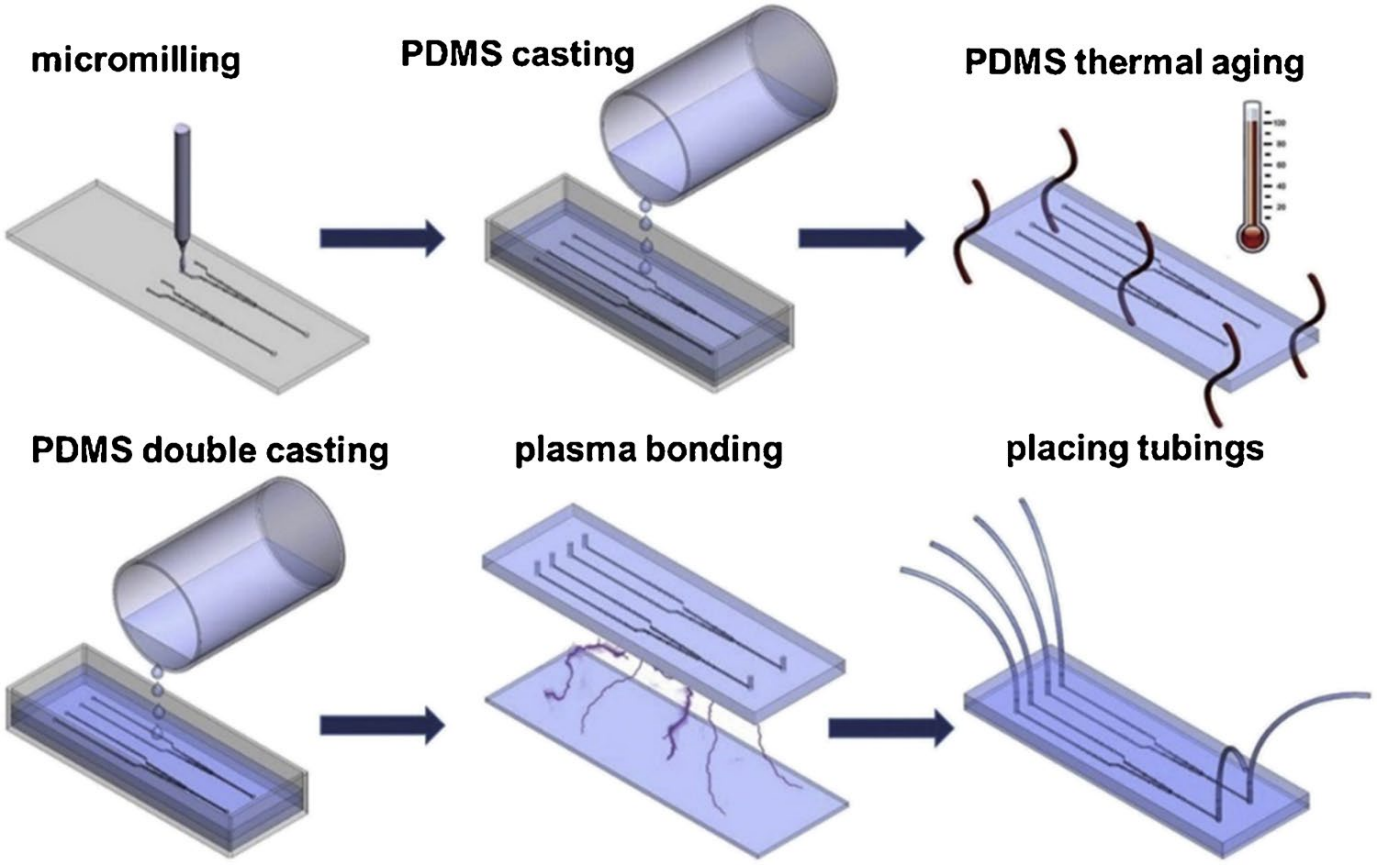
Lab-on-chip platform fabrication steps [72]

Tokarska K. et al. (Tokarska 2019) also worked on nano-photosensitizers to perform photodynamic therapy directly on the chip. The nano-PS fabrication guideline is presented in detail in the source article [72]. The biocompatible nano-PS consisted of multilayer capsules loaded with tetraphenyl porphyrin with an oil core. The microfluidic LOC platform was fabricated out of PDMS and glass layers (Fig. 21). The red LED (625 nm) was used as a light source during PDT. To investigate the biological activity of cells cultured in the microdevice, A549 human lung adenocarcinoma epithelial cells, and MRC-5 human fetal lung fibroblasts were selected. To test cellular responses and relationships after PDT, co-cultures of fibroblasts and pathological cells were used (such dual cell culture allowed for better mimicking in vivo conditions). According to the research results, the selective effect of the photosensitizer was proven and PS accumulated mainly in tumor cells. To test the cytotoxicity of free and encapsulated tetraphenyl porphyrin (PS) in the dark or after exposure to red LED light, cell viability assays were performed. Cells unexposed to PS constituted the control group. Cancer cells in the presence of encapsulated nano-PS in the dark showed the greatest loss of cell viability at a concentration of 20 µM. Fibroblasts also showed dose-dependent toxicity in the dark, but it was much lower than that of A549 cells. According to the research after illumination processes, while using tetraphenyl porphyrin in free form, no cellular phototoxicity occurred. On the other hand, nano-PS significantly affected the phototoxic effect, reducing the viability of the neoplastic cells to about 55%. In the case of MRC-5 fibroblasts, no toxicity was detected in any range of nano-photosensitizer concentrations. Reactive oxygen species that are destructive to cells were generated when tested with A-549 cancer cells. Moreover, more efficient ROS generation occurred with nano-PS than with the free tetraphenyl porphyrin photosensitizer. The highest phototoxicity was proven at a nano-PS concentration of 30 µM in monoculture. However, slightly lower phototoxicity was found for A549 in double cell culture, which can be the result of the presence of healthy fibroblasts nearby. To summarize, the developed and fabricated nano-photosensitizers were characterized by selective accumulation in cancer cells, high biocompatibility, and stability [72].

Summarizing this section, it is worth noting that the variability of spheroid formation each time can lead to inconsistent PDT results and complicated data interpretation. Moreover, although 3D spheroids are more reflective of the tumor environment than 2D cell cultures, they still do not fully reflect the complexity of actual tumor lesions.

## Conclusions

As mentioned in the “Introduction” section, the incidence of cancer globally is increasing year by year, and the worsening epidemiological situation of cancer has directed the scientific world to develop innovative cancer research methods. In this context, a comprehensive collection of scientific articles is presented relating to the future development of photodynamic therapy as a non-invasive approach to cancer cell research conducted directly on-chip. The concept combines theoretical and practical knowledge of mechanics, biomedical engineering, microsystems, and molecular and cellular biology.

To summarize the above content, lab-on-chip platforms supported by hydrogel matrices are excellent solutions for in vitro and ex vivo research. Biocompatible hydrogels mimic the three-dimensional cellular environment, such as the extracellular matrix, in which cells develop, differentiate, proliferate, and migrate. Therefore, a suitable hydrogel is an excellent environment for living cells because it replicates their natural conditions. Hydrogels could be considered cell transporters (more common) or structural parts of microdevices [20, 24]. The second application refers precisely to lab-on-chip platforms. The appropriate structure of such microchips, i.e., the presence of an ECM-like hydrogel environment (3D culture) and the presence of microchannels and microchambers allow for cell culture and transport of relevant substances. An allegory can be seen for the functioning of capillaries in a living organism and microchannels in LOC, where in both cases they perform transport-like functions. In addition, the ability to culture cells three-dimensionally enables cell–cell and cell-extracellular interactions, which are limited in two-dimensional cultures [13].

Moreover, a rapidly growing sector of science is focused on the additive manufacturing of hydrogels. Both natural and synthetic inks for 3D printing should exhibit adequate rheological properties to enable biomaterial printability. Furthermore, the hydrogels themselves must exhibit satisfactory mechanical and biological properties regarding hydrogel matrix strength and cell culture. According to ASTM standards, the following main technologies are distinguished in AM: extrusion-based 3D printing, jetting-based 3D printing, and vat photopolymerization-based 3D printing. According to the literature review, the extrusion technique is the most popular. Nevertheless, it presents a lower resolution and quite low printing speed in comparison with other technologies. The main advantages of extrusion are wide access to 3D printers and biocompatible inks and lower costs of the procedure. That is why, the extrusion of the ink is more widespread than the aforementioned techniques [17].

The 3D printing of LOC parts makes it possible to speed up microchip production, simplify the manufacturing procedure, and sometimes reduce the cost of the process, for example, by extruding the self-developed inks or bioinks. It should be highlighted that AM allows for the fabrication of complex, intricate structures that are often necessary for lab-on-chip devices. It may enable the design of customized geometries, including microchannels, microchambers, and integrated functional components tailored to specific applications. Moreover, AM facilitates rapid prototyping, allowing for quick iterations during development. This capability is particularly advantageous in research and development, where design changes can be easily implemented without the need for expensive and time-consuming tooling. Additive manufacturing enables the simultaneous use of different materials, especially hydrogels, but also other polymers and conductive inks. This capability is crucial for developing multifunctional LOC platforms with integrated sensors, actuators, and other components. For small-scale fabrication, AM is more cost-effective than traditional manufacturing methods. It eliminates the need for expensive molds and reduces material waste, making it ideal for producing small batches of customized LOC devices. Despite the many advantages of using additive manufacturing for hydrogel-assisted lab-on-chip platforms, several limitations could be mentioned. Despite the versatility of AM, the range of materials that can be used, particularly in hydrogel-assisted applications, is still limited. Not all hydrogels are suitable for 3D printing, and the mechanical properties of printed hydrogels can sometimes be inferior to those produced by traditional methods. Also restrictive are the requirements for the rheological properties of the hydrogel material constituting the ink concerning 3D printing. The resolution of AM processes may not always meet the stringent requirements needed for certain LOC applications, especially those requiring ultra-fine features at the microscale. Additionally, the post-processing of 3D-printed components may require more complex and time-consuming operations. Ensuring the biocompatibility and long-term stability of 3D-printed microfluidic devices, especially those incorporating hydrogels, can be challenging. Some 3D printing materials can degrade over time, affecting the performance and safety of the devices. Furthermore, the most presented research focused primarily on small-scale constructs. Scaling up to clinically relevant sizes while maintaining structural integrity and cell distribution remains challenging.

As mentioned in the paper, hydrogels can be enriched with functional additives, such as carbon nanotubes. The CNTs mimic collagen due to their fibrous form and similar dimensions. Because of the CNTs biocompatibility, they can be combined with several natural hydrogels to perform diverse biological studies. In addition to biocompatibility, the combination of hydrogels and CNT also improves mechanical stability, cell adhesion, and development [64]. Moreover, the inclusion of CNTs in the hydrogel structure is neutral for the AM processes [65].

One of the first papers focusing on photodynamic therapy performed in a microfluidic system was published by Polish researchers (E. Jędrych et al., 2011). The researchers compared the results of PDT for macroscale (Petri dish) and microscale (lab-on-chip) cell cultures. The efficacy of this particular anti-cancer therapy was comparable in both cases and as a result, the authors proved the validity of using microfluidic platforms in subsequent tests and optimizations of PDT processes. This ensured, i.e., a reduction of manufacturing costs and the development of anti-cancer therapies [70]. Since then, many innovative and future-oriented solutions (examples: [16, 71]) for performing PDT directly on the microchip have been developed and presented to the scientific world.

The content of this review article makes up the whole, gathering knowledge in the development of anti-cancer therapies (concerning technical and biological aspects) and raising the level of skills in biomedical research.

## Data Availability

No datasets were generated or analysed during the current study.
